# Trait modeling to predict benthic functions and vulnerabilities across black sea seascapes

**DOI:** 10.1038/s41598-025-24508-4

**Published:** 2025-11-07

**Authors:** Séverine Chevalier, Olivier Beauchard, Luc Vandenbulcke, Adrian Teaca, Tatiana Begun, Valentina Todorova, Karline Soetaert, Marilaure Grégoire

**Affiliations:** 1 Modelling for Aquatic Systems (MAST) - Research Unit FOCUS, University of Liège, Liège, Belgium; 2 Ghent University, Ghent, Belgium; 3 Department of Estuarine and Delta Systems (EDS), Royal Netherlands Institute for Sea Research, Yerseke, Netherlands; 4 Department of Biology and Ecology, National Research and Development Institute for Marine Geology and Geoecology (GeoEcoMar), Bucharest, Romania; 5 Institute of Oceanology - Bulgarian Academy of Sciences (IO-BAS), Varna, Bulgaria

**Keywords:** Trait distribution model, Macrobenthos, Northwestern shelf of the black sea, Marine functional biodiversity, Ecosystem functions, Spatial mapping, Ecology, Ecology, Environmental sciences, Ocean sciences

## Abstract

Benthic biodiversity is of global significance for the provision of ecosystem services and the mediation of global biogeochemical cycles. The lack of detailed spatial distributions of the functions and vulnerabilities of the benthos critically prevents us from protecting benthic biodiversity and its functioning in the context of increasing human perturbations and climate change. Here, we propose a multidisciplinary approach to bridging in situ benthic data to the maps of macrobenthic functions and vulnerabilities at the scale of the northwestern shelf of the Black Sea. Our findings show that oxygen availability is a key driver of the functional trait composition of macrozoobenthic communities. Shallower well-oxygenated areas support high biomixing and bioirrigation on muddier-sandier substrata and high biodeposition on coarser substrata associated with mussel reef communities. In contrast, at depleted oxygen areas at the edge of the shelf, macrobenthic communities are functionally impoverished with only a combination of a few typical opportunistic traits, and those communities have a negligible impact on ecosystem functions. Mapping of vulnerabilities and functions of benthic communities can support marine management strategies aligned with the United Nations Sustainable Development Goal 14: Life Below Water.

**Supplementary Information:**

The online version contains supplementary material available at 10.1038/s41598-025-24508-4.

## Introduction

Macrozoobenthic communities inhabiting sediments has an important impact on sediment geochemistry and global biogeochemical cycles^1–3^. During their movement, feeding, respiration and burrowing activities, benthic animals rework sediment and move water (ventilation) in a process termed bioturbation^1,4^. Bioturbation impacts the upward and downward transports of particles through biomixing, and increases the exchange of solutes with the overlying water through bioirrigation^4,5^. Through these ecological processes, macrofauna alter the physical and chemical properties of sediments, modify microbially driven reactions, influence the resources for other biota and promote the oxygenation of the sediment, leading to greater mineralization and nutrient turnover^1,6,7^. Filter-feeders can also play a role in enriching the sediment by filtering particles from the overlying water and excreting nondigestible materials such as feces and pseudofeces (i.e., biodeposits) at the sediment-water interface^8^. In this process, termed biodeposition, carbon and nitrogen-enriched deposits provide food for other organisms and stimulate microbial production^9,10^. At the scale of the ecosystem, bioturbation by benthic macrofaunal communities has a great impact on marine ecosystem functions such as recycling and storage of organic carbon and other elements (e.g., removal of nitrogen through denitrification) in shallow coastal habitats^11–13^.

Macrozoobenthos is thus a dominant actor in biogeochemical cycles and ecosystem functioning and is currently threatened by global environmental changes and human activities at sea. Climate change is foreseen to cause major changes in marine habitats, through increased water temperature, ocean acidification and the expansion of oxygen minimum zones, all of which will alter the health of benthic life, threatening its functioning and its ability to underpin the delivery of ecosystem services^14^. The removal of ecosystem engineers, such as bioturbators, could induce large changes in the structure of the habitat, with cascading effects on local biodiversity and impairing the macrozoobenthos contribution to ecosystem functioning^2,15,16^.

The vulnerabilities of benthos to disturbances and their role in ecosystem functioning can be estimated by the traits of the species. Species traits are the morphological, phenological and physiological characteristics of a species^17^. According to the theory of Southwood^18^, species traits are strongly related to the environment more than the species composition. This trait-environment link can be used to develop trait distribution models to upscale very sparse measurements in the benthic system across ecosystem scale. Benthic functional maps are essential for conducting integrated ecosystem assessments^19^ and bridging in situ local data to the scale of ecosystem-based management^15,20^. However, the mapping of ocean biology and, in particular, of benthic biology, is still far behind that of terrestrial systems^21,22^. In terrestrial ecology, the development of correlative bioclimatic models or niche-based ecological models that assess the distribution of a given species based on the distribution of the environmental variables to which it is suited has been accelerated by the necessity of assessing the effect of climate change on biodiversity^23^. This development has also been favored by the significant progress in our ability to observe the Earth’s surface with increased spatial and temporal resolution^24,25^. However, benthic system is still clearly undersampled, especially for biological and environmental data^26,27^. Benthic sampling is strongly heterogeneous and it is very rare to have the necessary data to develop mapping tools (i.e., biological and environmental data sampled at the same place and time to assess the species niche^28^. This lack of data has greatly prevented regular mapping of the benthic system. There are still very few maps of the benthos species, functions and biodiversity^21,29^. This limitation hampers sound management and modeling of the benthic system in relation to climate change and biodiversity protection^30^. As a result, current ocean numerical models ignore the variability of life on the seafloor and its impact on biogeochemical cycles and ecosystem functioning^15,20^. In recent years, there has been a change in the paradigm concerning the availability of environmental data for the ocean^31^. Since 2014, the European Marine Copernicus Service (CMEMS) has provided daily 10-day forecasts and 20–30 years of reanalysis of the physical and biogeochemical states of the global and Arctic oceans and European regional seas (marine.copernicus.eu). These modeled environmental data combined with in situ data can remedy the lack of available environmental maps to support the development of mapping methods for the benthos.

In this paper, we study the relationships between traits of macrozoobenthos and environmental conditions. Relevant functional traits are selected, and their connections with environmental conditions, both in situ measured and modeled, are analyzed. This analysis is done for individual traits and for combinations of traits that define empirical indicators of ecological processes or vulnerabilities. The trait-environment relationships are investigated at the local scale and are subsequently scaled up to obtain maps at the ecosystem scale of individual traits and derived indicators of ecological functions. These maps can be used to support management strategies (e.g., definition of marine protected areas) and the inclusion of benthic life characteristics in ocean numerical models. This study was performed on the northwestern shelf of the Black Sea, which features an important gradient in species trait composition and environmental conditions due to the large river discharge and the strong gradient in bottom-water oxygen concentrations.

## Results

### Functional diversity

Our study identifies three dominant life history strategies based on response traits: “P”- precocial with short life cycle and high offspring survival probability, “O”- opportunist with a relatively short life span and low offspring survival probability and “E”- episodic with a longer longevity for achieving a minimum of reproductive success. A more complete description of the three groups is provided in the Supplementary Information.

**Fig. 1:**
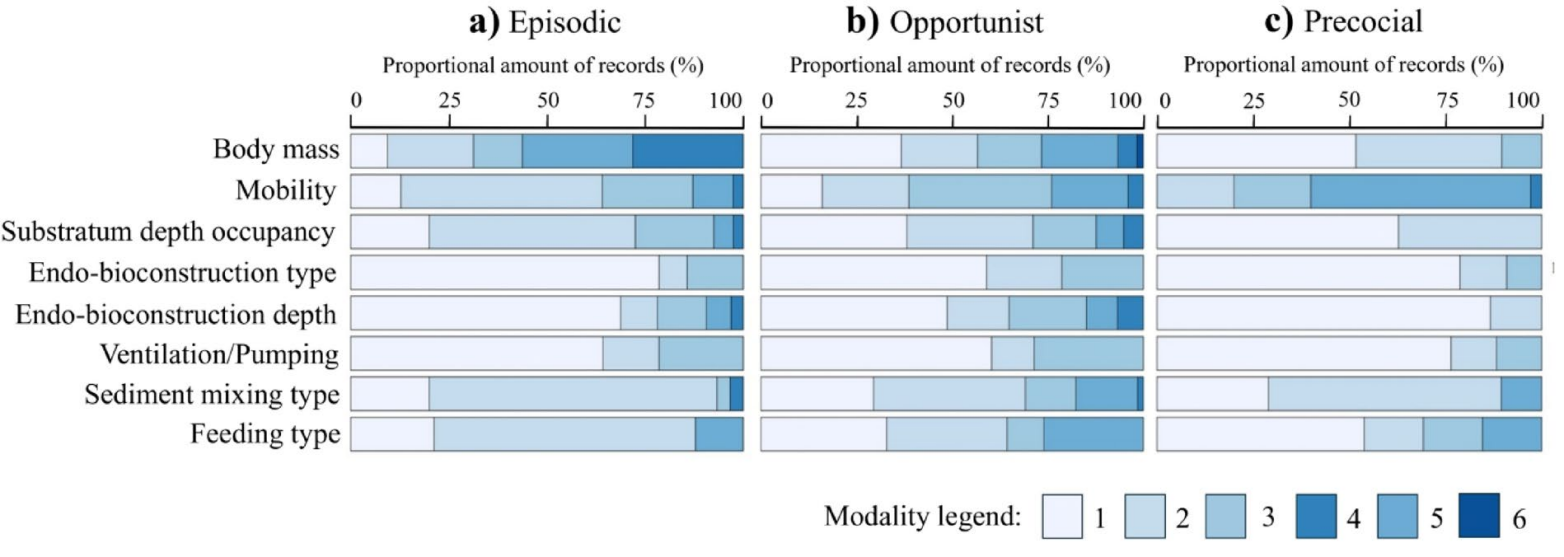
Relative amounts of records within effect traits per life history group: (**a**) “E”- episodic species, (**b**) “O”- opportunist species and (**c**) “P”- precocial species. The modality codes correspond to those in Table 2.

**Fig. 2:**
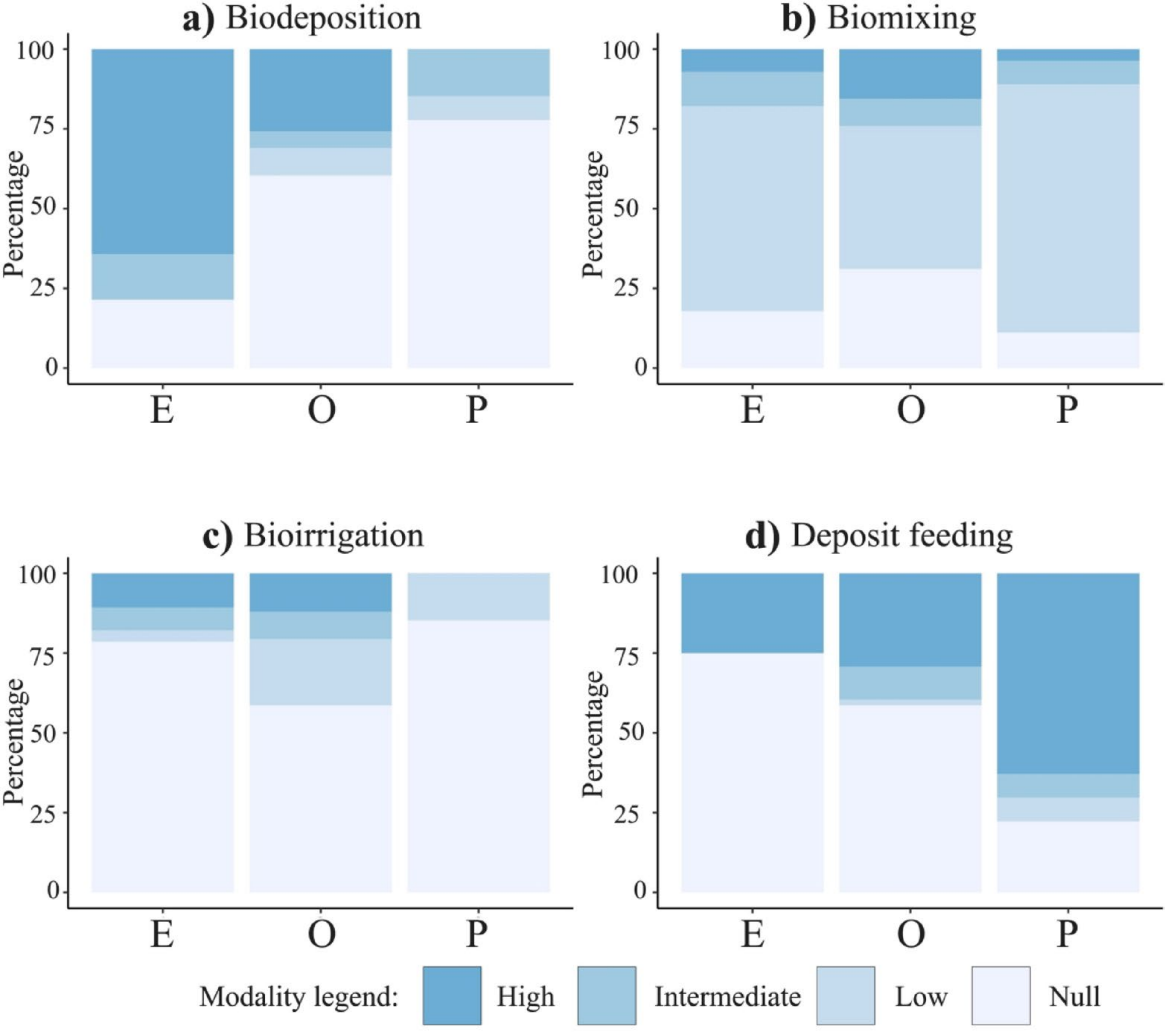
Contribution to the four ecological indicators: (**a**) biodeposition, (**b**) biomixing, (**c**) bioirrigation and (**d**) deposit feeding, defined in Table 3) at the species level per life history group (“E”- episodic, “O”- opportunist and “P”- precocial).These indicators are classified into four categories: null (0), low (0 to 0.1), intermediate (0.1 to 0.2) and high (> 0.2). The color bar on the bottom represents, from light blue to dark blue, increasing ecological indicator scores.

The three life history groups exhibit distinct combinations of effect traits (Fig. 1a-c), reflecting different ecological roles (Fig. 2a-d). Most species occupy the upper 5 cm of sediment, with no ventilation or bioconstruction, and are primarily biodiffusors (Fig. 1a-c). Episodic species are mainly suspension feeders, often epifaunal, with limited mobility and no bioconstruction (Fig. 1a). The opportunistic group includes the highest proportion of deep burrowers, greatest ventilation activity, complex endo-bioconstruction structures, and upward or downward conveyors (Fig. 1b). Precocial species are mobile deposit feeders, mostly epifaunal or shallow burrowers, with biodiffusive or downward conveying activity (Fig. 1c).

These differences in effect traits are directly reflected in the ecological indicators derived from those traits. Higher biodeposition scores are mostly associated with episodic, along with opportunist and precocial species (Fig. 2a). The highest scores for biomixing and bioirrigation are associated with opportunist followed by episodic species (Fig. 2b, c). Most species are characterized by low biomixing potential, regardless of their life history group (Fig. 2b). For bioirrigation, a large proportion of the species have a null contribution, especially for the episodic and precocial groups (Fig. 2c). Precocial deposit feeding scores are greater than episodic scores (Fig. 2d).

### Relationships between traits and abiotic descriptors through RLQ analysis

#### Life history strategies

The RLQ analysis is not significant; however, testing correlations between traits and abiotic scores with the RLQ axes revealed that the first axis is significantly correlated with the “Precocial” and “Opportunist” strategies, but not with the content of particulate organic carbon in the sediment.

**Fig. 3:**
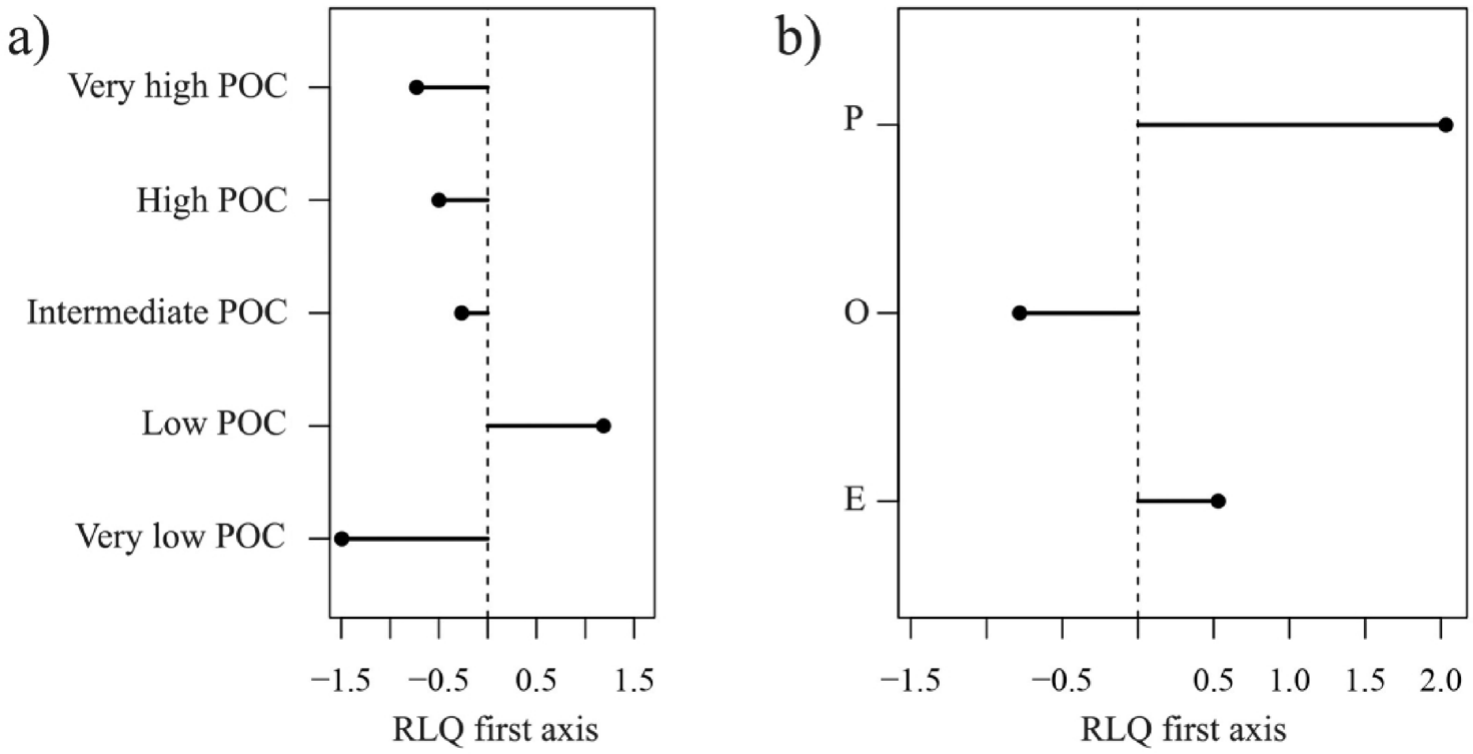
(**a**) Abiotic descriptor scores and (**b**) life history strategy scores along the first axis of the RLQ analysis. The first axis accounts for 91% of the total variability of the RLQ.

On the first axis of the RLQ analysis, the group opportunist is associated either with (very) high or very low content of organic carbon (POC) either at very shallow coastal or very deep stations (Fig. 3a, negative values on the first axis). Precocial and episodic strategies are associated with lower POC at shallow to intermediate water depths (Fig. 3b, positive values). The spatial distribution of sampling station scores on RLQ axes is presented in the Supplementary Information. Further details on the RLQ analysis, including its significance and the combination with the fourth-corner method, are also available in the Supplementary Information.

#### Response traits

The RLQ analysis evidences a globally significant correlation between the selected traits and environmental variables. The first two axes of the RLQ are significantly correlated with sexuality, mobility and offspring development, as well as oxygen and salinity.

**Fig. 4:**
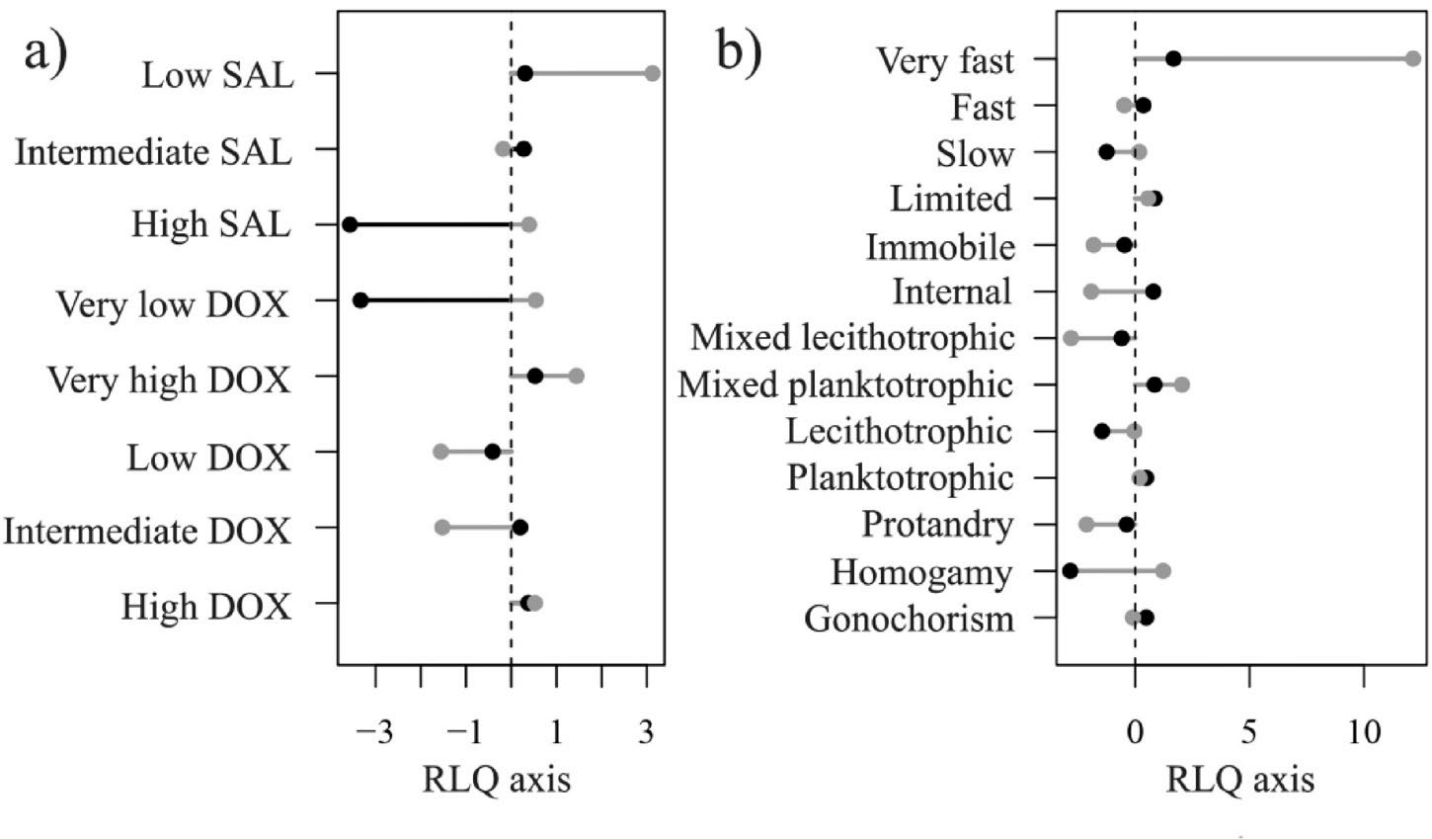
(**a**) Abiotic descriptor scores and (**b**) modalities scores on the first axis of the RLQ (black) and the second axis of the RLQ (grey). The first axis accounts for 90% of the total variability of the RLQ and the second axis for 8%.

**Fig. 5:**
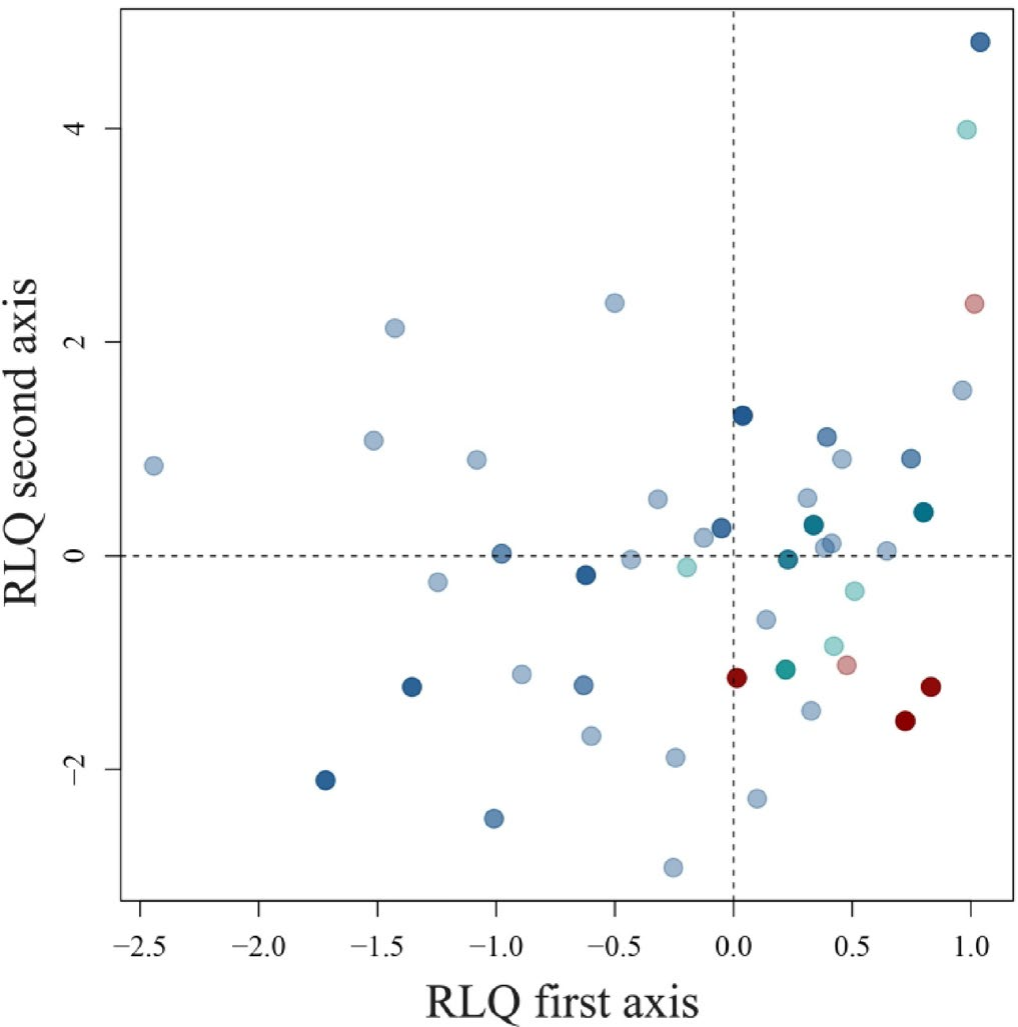
Species scores on the two first axes of the RLQ. Each dot corresponds to a species. Each color corresponds to a life history group, with dark red for Precocial, dark blue for Opportunist, and dark cyan for Episodic. The intensity of colors varies depending on the number of dots superimposed. A darker intensity of color indicates that two or more taxa share the same scores on both axes of the RLQ. A more detailed representation of species scores per life history group is provided in the Supplementary Information.

The first axis of the RLQ is associated with a depth-gradient: low oxygen and high salinity values (typical of shelf break conditions) are found in the negative part, while higher oxygen content and lower salinity are found in the positive part (Fig. 4a). In contrast to shallower well-oxygenated waters, which include species from the three life history groups with multiple modalities of traits, deep hypoxic stations are inhabited only by opportunists (Figs. 4b and 5).

The second axis of the RLQ differentiates the stations based on their depths and oxygen levels. In shallow and well-oxygenated waters (positive scores on both axes, Fig. 4a), we find either mixed-planktotrophic development, very fast mobility or limited mobility (Fig. 4b). On positive scores on the second axis, there are also very deep hypoxic stations with only some opportunists with slow mobility, homogamy and (mixed)- lecithotrophic development (Figs. 4b and 5). At intermediate depths and oxygen levels (positive and negative scores for the first and second axes, respectively, Fig. 4a), we show either fast species with an internal mode of development (typical of precocial strategists, Fig. 5) or species with no mobility such as episodic bivalves or fixed opportunist worms (Figs. 4b and 5). At deeper depths and lower oxygen contents, (negative scores on both axes, Fig. 4a), we find mostly opportunistic species (Fig. 5) with protandry, mixed- lecithotrophic development and no mobility (Fig. 4b). The spatial distribution of sampling station scores on RLQ axes is presented in the Supplementary Information. Further details on the RLQ analysis, including its significance and the combination with the fourth-corner method, are also available in the Supplementary Information.

#### Effect traits

The global RLQ analysis is not significant; however, testing correlations between traits and abiotic scores with the RLQ axes shows significant correlation between the first axis of the RLQ and substratum depth occupancy, ventilation/pumping ability and substratum. The second axis is correlated only with salinity and ventilation/pumping ability.

**Fig. 6:**
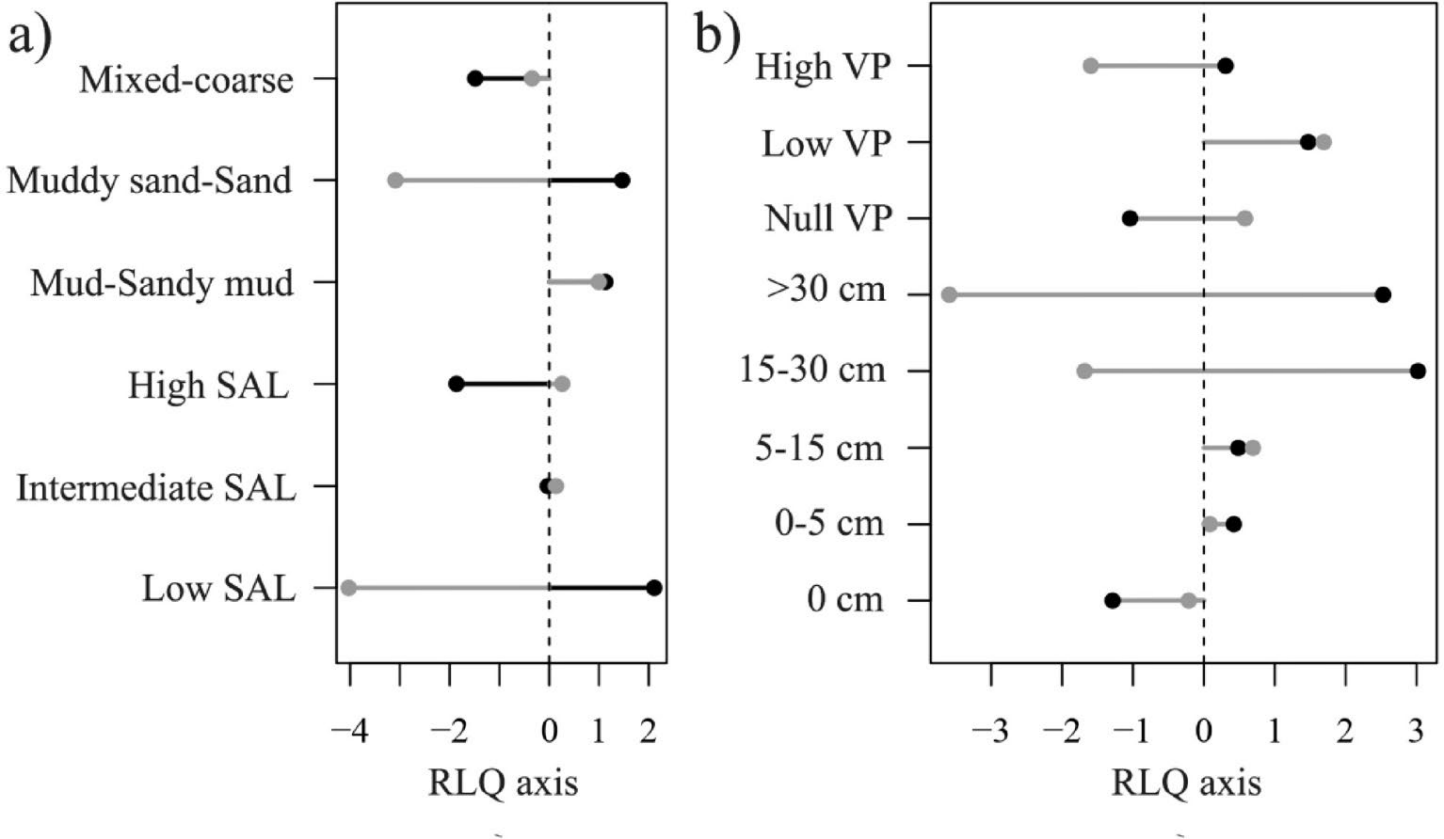
(**a**) Abiotic descriptor scores and (**b**) modalities scores on the first axis of the RLQ (black) and the second axis of the RLQ (grey). The first axis accounts for 67% of the total variability of the RLQ and the second axis for 32%.

The first axis of the RLQ analysis separates mixed-coarse substrata at intermediate to high salinity with epibenthos and no ventilation/pumping (negative values, Fig. 6a, b) from the finer substrata, at low to intermediate salinity with endobenthic species with ventilation/pumping activities (positive values, Fig. 6a, b). The second axis separates low-salinity sites with higher ventilation pumping from intermediate to high salinity sites with lower ventilation/pumping ability (Fig. 6a, b). The spatial distribution of sampling station scores on RLQ axes is presented in the Supplementary Information. Further details on the RLQ analysis, including its significance and the combination with the fourth-corner method, are also available in the Supplementary Information.

#### Ecological indicators

**Fig. 7:**
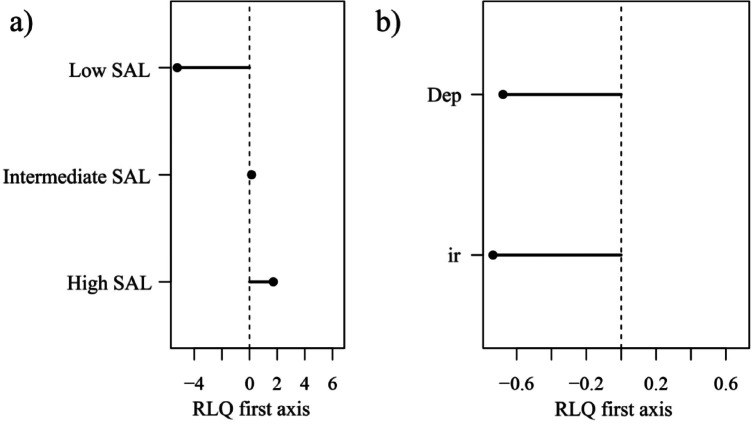
(**a**) Abiotic descriptor scores and (**b**) ecological indicator scores on the first axis of the RLQ. The first axis accounts for 99.99% of the total variability of the RLQ and the second axis for 0.01%.

The RLQ analysis is not significant; however, testing correlations between traits and abiotic scores with RLQ axes, show that the first axis of the RLQ is significantly correlated with bioirrigation and deposit feeding potential, as well as salinity. On the first axis of the RLQ analysis, higher intensity of bioirrigation and deposit-feeding potential are associated with low salinity (Fig. 7a, b). The spatial distribution of sampling station scores on RLQ axes is presented in the Supplementary Information. Further details on the RLQ analysis, including its significance and the combination with the fourth-corner method, are also available in the Supplementary Information.

### Mapping of traits

#### Spatial distribution of life history groups

**Fig. 8:**
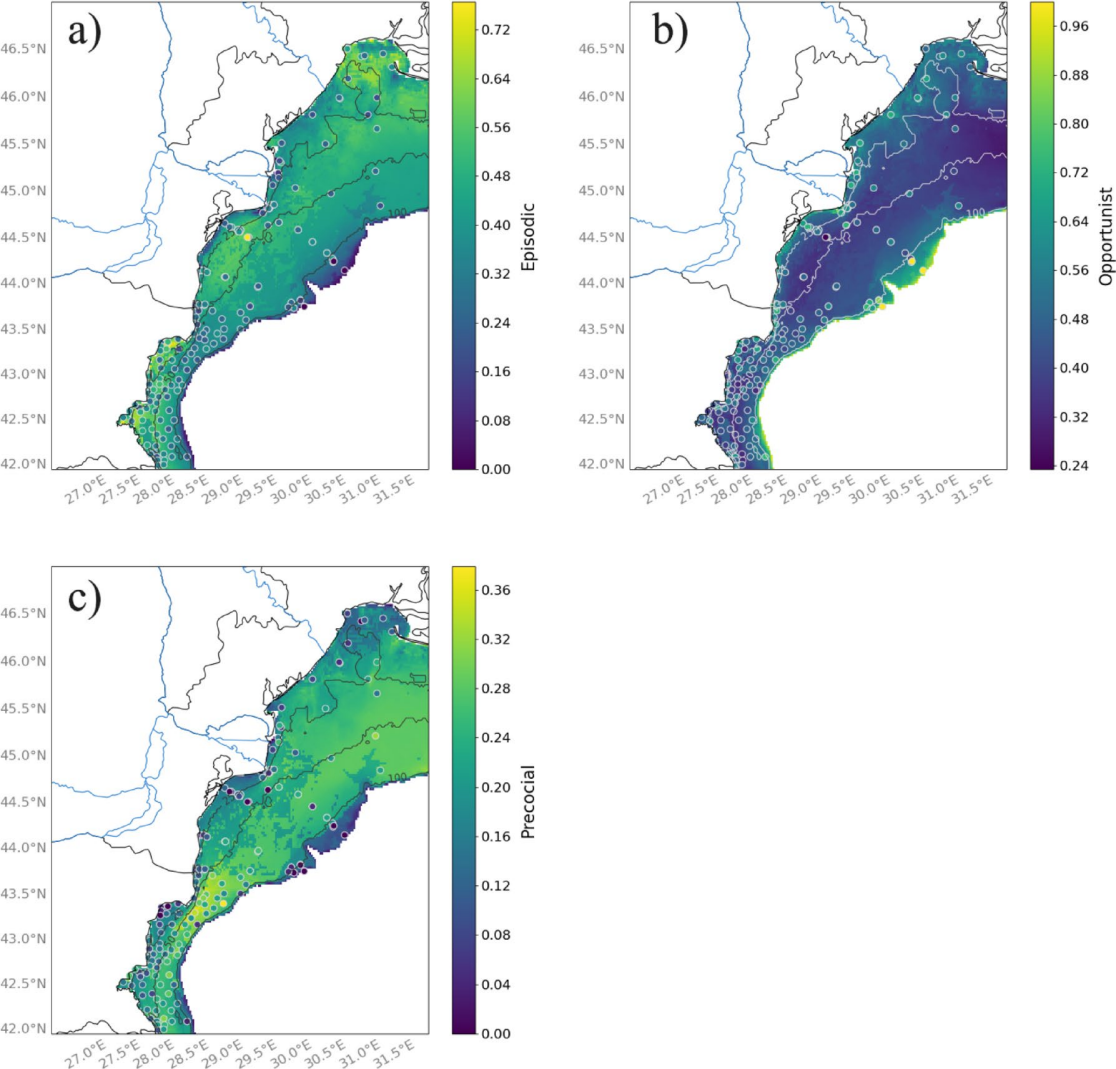
Spatial distribution of the three life history strategies across the northwestern shelf of the Black Sea: (**a**) episodic, (**b**) opportunist and (**c**) precocial species. Dots represent sampling stations colored according to the measured proportion of life history strategies within a community. The color bar on the left ranges from dark purple (low values) to yellow (high values). Blue lines indicate rivers, black and white lines show bathymetry, and areas deeper than 200 m are colored in white. Color bar scales differ between maps and are not directly comparable. Maps were produced with the Cartopy^81^ package (v0.24.1) in the open-source software Python 3.12. The Python software is available from https://www.python.org/downloads/. The Cartopy package is available at https://scitools.org.uk/cartopy.

The distribution of episodic species is higher in the southern part and near the mouth of the Dnieper River in the upper northeastern sector (Fig. 8a). At the edge of the shelf, the benthic communities are dominated by opportunistic species (Fig. 8b). The proportion of opportunists is also slightly greater in coastal areas close to the Danube mouth and in the northeastern part of the shelf (Fig. 8b). The proportion of precocial species is higher at intermediate depths, particularly in the southern part of the shelf (Fig. 8c).

#### Spatial distribution of response traits

**Fig. 9:**
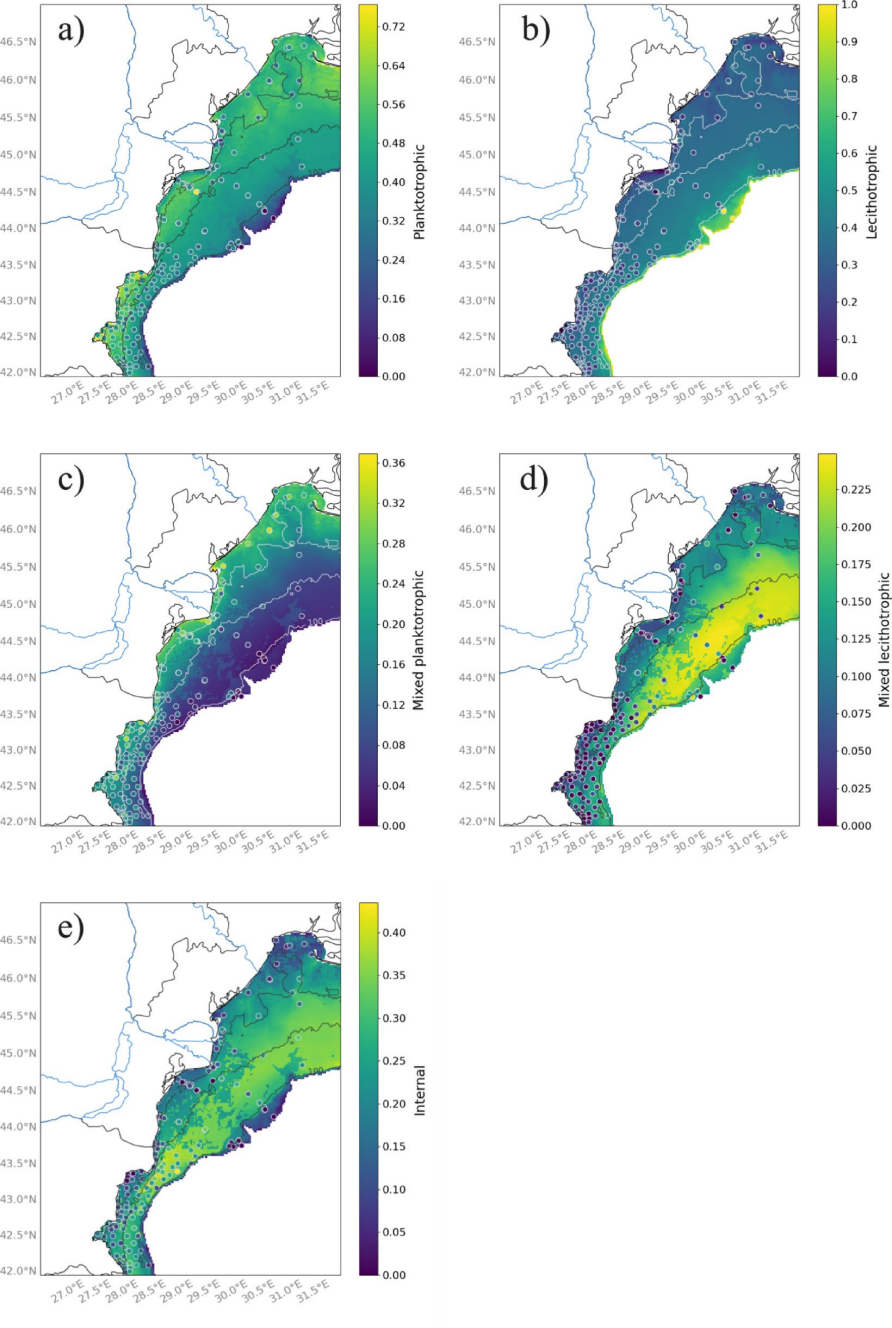
Spatial distribution of the five modalities of trait offspring development across the northwestern shelf of the Black Sea: (**a**) planktotrophic, (**b**) lecithotrophic, (**c**) mixed-planktotrophic, (**d**) mixed-lecithotrophic and (**e**) internal. Dots represent sampling stations colored according to the measured proportion of modalities of offspring development within a community. The color bar on the left ranges from dark purple (low values) to yellow (high values). Blue lines indicate rivers, black and white lines show bathymetry, and areas deeper than 200 m are colored in white. Color bar scales differ between maps and are not directly comparable. Maps were produced with the Cartopy^81^ package (v0.24.1) in the open-source software Python 3.12. The Python software is available from https://www.python.org/downloads/. The Cartopy package is available at https://scitools.org.uk/cartopy.

The planktotrophic and mixed planktotrophic modes decrease along a depth gradient (Figs. 9a? c). In contrast, the proportions of lecithotrophic and mixed lecithotrophic mode are higher at the edge of the shelf (Figs. 9b, d). The spatial distribution of internal development (Fig. 9e) follows the same pattern as that of the precocial group (Fig. 8c), as this mode of development is almost exclusively found in this group.

#### Spatial distribution of effect traits

**Fig. 10:**
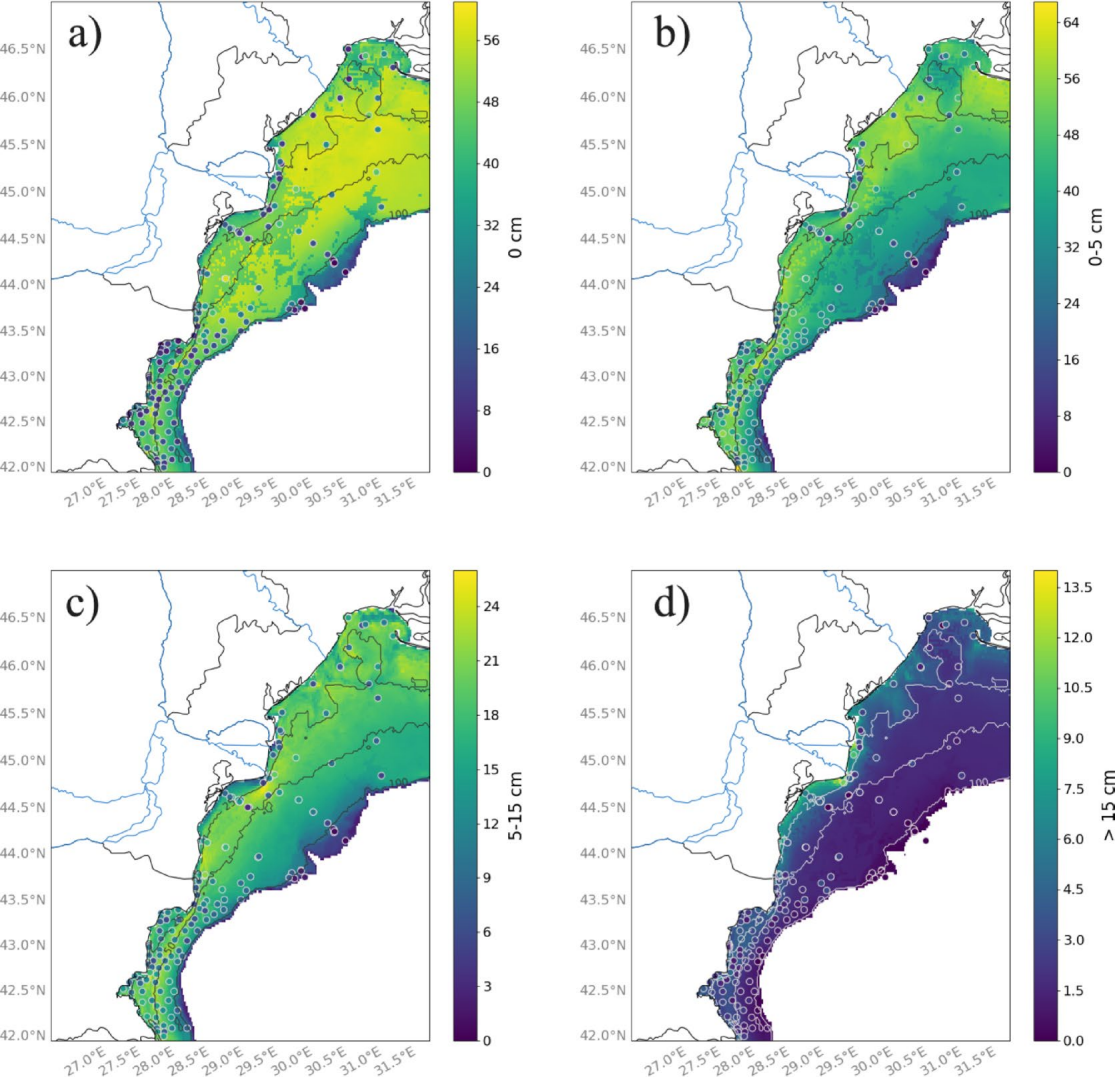
Spatial distribution of the modalities of trait substratum depth occupancy (in cm) across the northwestern shelf of the Black Sea: (**a**) 0 cm, (**b**) 0–5 cm, (**c**) 5–15 cm and (**d**) deeper than 15 cm in the sediment. Dots represent sampling stations colored according to the measured intensity of modalities of substratum depth occupancy within a community. The color bar on the left ranges from dark purple (low values) to yellow (high values). Blue lines indicate rivers, black and white lines show bathymetry, and areas deeper than 200 m are colored in white. Color bar scales differ between maps and are not directly comparable. Maps were produced with the Cartopy^81^ package (v0.24.1) in the open-source software Python 3.12. The Python software is available from https://www.python.org/downloads/. The Cartopy package is available at https://scitools.org.uk/cartopy.

The epifaunal distribution (i.e., 0 cm) is slightly higher at deeper water depths and lower in the area close to the mouth of the Danube Delta (Fig. 10a). Other modalities (i.e., 0–5 cm and 5–15 cm) have less clear spatial patterns, but the proportion of burrowing species (from 0 to 15 cm) is, in general, higher at shallower coastal areas (Figs. 10b, c). Very deep burrowers (i.e., > 15 cm) are mainly distributed near the coast close to the Danube Delta (Fig. 10d).

#### Spatial distribution of the ecological indicators

**Fig. 11:**
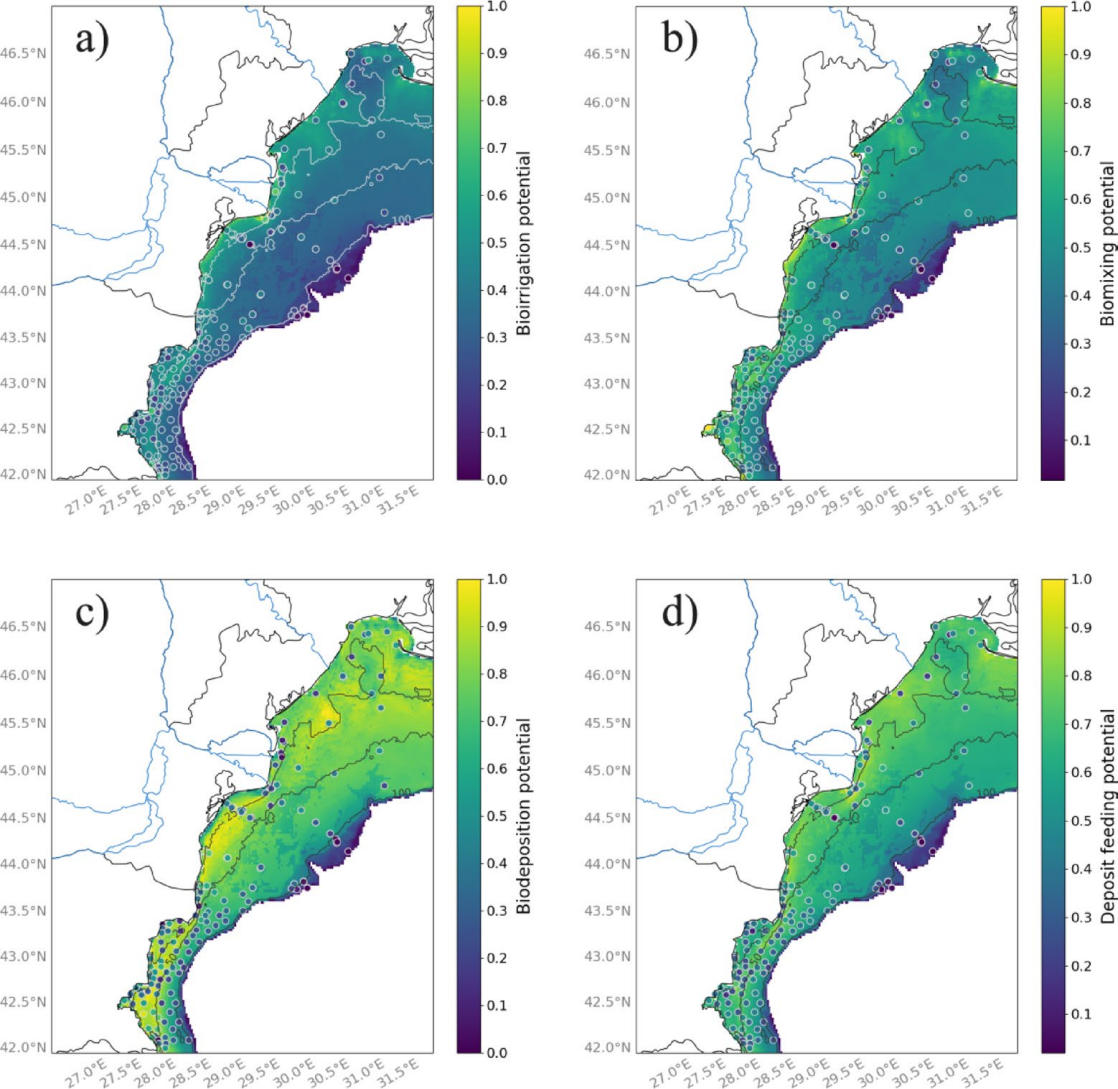
Spatial distribution of the ecological process indicators across the northwestern shelf of the Black Sea: (**a**) bioirrigation, (**b**) biomixing, (**c**) biodepostion and (**d**) deposit feeding potentials. Dots represent sampling stations colored according to the measured intensity of ecological processes within a community. The color bar on the left ranges from dark purple (low values) to yellow (high values). Blue lines indicate rivers, black and white lines show bathymetry, and areas deeper than 200 m are colored in white. Color bar scales differ between maps and are not directly comparable. Maps were produced with the Cartopy^81^ package (v0.24.1) in the open-source software Python 3.12. The Python software is available from https://www.python.org/downloads/. The Cartopy package is available at https://scitools.org.uk/cartopy.

The four community ecological indicators exhibit similar spatial pattern across the shelf, with higher intensities occurring in shallower oxic waters and decreasing to zero at the edge of the shelf (Fig. 11a-d). This finding aligns with the RLQ analysis, as higher intensities of bioirrigation and deposit feeding were linked to very shallow low-salinity sites (Fig. 7a, b). Higher bioirrigation potential is observed within a narrower coastal band (Fig. 11a) compared to biomixing potential, with a more scattered distribution towards deeper depth (Fig. 11b).We observe hotspots of biodeposition near the Dnieper River Delta in the northeastern region or in the coastal southern area of the shelf (Fig. 11c). Deposit feeding potential is generally high across the whole shelf except in very deep area (Fig. 11d).

## Discussion

One of the key challenges in biodiversity conservation is to upscale local sub-meter ecological processes to a broader scale, which is relevant for ecosystem-based management^15,20^. The mapping of ocean biodiversity lags far behind that of terrestrial ecosystems; one of the reasons is the lack of ocean biological and environmental information. For the past decade, quality-controlled physical and biogeochemical data for all European regional seas and the open ocean have been freely available through the European Marine Copernicus Service (CMEMS: marine.copernicus.eu). This opens doors to the development of biogeographic models providing the necessary scientific framework for mapping marine species, traits and functions at ecosystem scales. These maps offer a solid support for the implementation of marine policy to preserve biodiversity (e.g., design of marine protected areas) and to allocate blue growth activities (e.g., marine spatial planning as requested by the recently established Intergovernmental Science Policy Platform on Biodiversity and Ecosystem Services (IPBES)).

This paper proposes a methodology to scale up local species data to obtain maps of ecological groups and functions at shelf scale. The methodology combines species-level trait assessments with the derivation of community-level ecological functions from these traits using empirical formulations. The link between the community traits and functions with environmental conditions was assessed using multivariate statistical approaches and machine learning. The mapping of the traits and functions is then performed using the environmental variables that are significantly correlated with the traits and that are predicted by a numerical ocean model. The methodology was developed for the northwestern shelf of the Black Sea, which constitutes an ideal test area because it hosts significant gradients of environmental conditions that are expected to affect trait composition and biodiversity. We focused on effect traits, which reflect the benthos’ influence on benthic-pelagic coupling and on response traits that characterize species sensitivity to environmental disturbance.

The distribution of the data covers the northwestern shelf area from the river side and coastal areas to offshore up to the shelf break. This allows us to investigate the trait-environment link across a large environmental gradient dominated by oxygen (well oxygenated, hypoxic and anoxic waters), salinity (fresh to marine waters), depth (from 10 up to 150 m), and substratum (muddy, sandy and coarser grained with shells).

We show that variability of environmental conditions, such as the gradient of bathymetry, bottom dissolved oxygen, sedimentary organic matter, and type of substratum, explain the spatial distribution of traits. In particular, the gradient of bathymetry, bottom dissolved oxygen, sedimentary organic matter and type of substratum are the environmental variables that explain the most of the variability in traits. The level of bottom oxygen is a dominant factor that explains the diversity of traits and the distribution of ecological groups: at very low oxygen levels, we find low functional biodiversity with only some opportunistic species, while in well-oxygenated waters, higher trait divergence is observed with a marked diversity of offspring and adult traits. In previous studies, it was found that trait composition changes dramatically as oxygen concentration decreases^32,33^, with only asexually reproducing polychaetes found in oxygen-deficient zones^34,35^.

Three of the four life history strategies proposed in^36^ are present in the Black Sea macrozoobenthos: opportunist, precocial and episodic. Half of the taxa are grouped as opportunists, which is consistent with historical disturbances, including widespread bottom hypoxia and eutrophication^37,38^. The opportunistic species are highly functionally heterogeneous, and their distribution is not habitat specific. They are mostly found in areas characterized by a high seasonality of oxygen, such as the northeastern part of the shelf, which has a high sedimentary organic matter content and seasonal hypoxia, and the shelf break where upwellings of anoxic waters occur from the deep basin. In these two distinct seascapes, opportunist species are separated according to their offspring development mode and mobility. In shallow, oxygen-rich areas, mixed-planktotrophic development is prevalent, consistently with the reproductive strategies of deep-burrower opportunist decapods. In contrast, mixed-lecithotrophic and lecithotrophic modes are associated with deeper, hypoxic environments and taxa such as opportunistic tubicolous worms (e.g., capitellids and spionids), which usually persist in disturbed environments^39^. We also show that homogamy is restricted to hypoxic zones, as this reproductive mode may confer advantages under sparse population densities, as documented in^40^.

The small fraction of the episodic group, which is only present in well-oxygenated areas, and the absence of survivors, indicate that the benthic communities are still in a phase of ecological recovery^41,42^. Planktotrophic development, which is typical of the group episodic, is most prevalent at shallow and intermediate depths. In shallower areas, episodic species are mostly shallow-burrowing clams with limited mobility in opposition to immobile epifaunal episodic species, such as mussels found at intermediate levels of oxygen and depth. There are also mobile species, such as epifaunal carnivorous worms or small precocial amphipods, documented to be hosted by biogenic reefs of mussels^43–45^.

The spatial distributions of ecological processes follow a clear environmental gradient with diverse functionally seascapes along a depth-gradient. Shallow coastal waters, with finer-grained substrata, support deep-burrowing taxa with high ventilation activity, leading to higher scores for both biomixing and bioirrigation. These habitats are also characterized by higher species densities and trait diversity, amplifying the contribution of macrobenthos to those ecological processes. At intermediate depths, epifaunal species with no ventilation or pumping abilities are associated with mixed and coarse substrata. Coarser sediments have already been shown to generally support more epifaunal species, whereas fine sandy sediments are more favorable for burrowers^46–48^. Despite their smaller contributions to biomixing or bioirrigation, large-bodied suspension feeders (e.g., bivalves, sponges, and ascidians) can significantly contribute to ecosystem functioning through biodeposition^8,49^. At greater depth, hypoxic shelf-edge zones are dominated by impoverished benthic communities with negligible impacts on ecosystem functions. Our finding support previous works evidencing that bioturbation declines along increasing hypoxic gradients^33,50,51^.

Our study has several limitations inherent to the sampling methods and available data. Firstly, the contribution of deep-dwelling taxa is likely underestimated by the Van Veen grab sampling method^52^. The resolution of the ocean model that delivers the environmental condition is at the km scale (i.e., 2.5 km), while that of the community is at the meter scale. This mismatch of spatial scales can explain the limited number of significant trait-environment relationships found in this study. Neural networks can help to deal with non-linearities in trait-environment relationships and give sufficiently good results in mapping the modalities of the traits or indicators. However, our model has limitations in predicting extreme values that are less often sampled. Finally, many trait-based indicators are empirical, and their validation is limited due to the lack of in situ data or laboratory experiments^53^. Several mathematical formulations for the same indicator have been proposed^54–56^. In this study, we focus on the effect traits that impact benthic-pelagic coupling. Combinations of these traits are used via empirical formulations to assess ecological functions such as biomixing, bioirrigation and biodeposition. The aim is to use the variability of these ecological indicators to account the effect of benthic life in ocean biogeochemical models. However, these indicators are not necessarily directly linked with biogeochemical model parameters^57^. For instance, an information on the variability of biomixing cannot be used straightforwardly in the parametrization of the sediment mixing layer depth. This highlights the potential benefit of mapping the modalities of traits as a better proxy. By mapping trait modalities, such as the sediment living depth, we are closer to the property measured at the species level and avoid the use of empirical equations to compute imperfect indicators, which are still poorly validated. The obtained trait maps can be used to incorporate the variability of benthos characteristics in the parametrization of ocean models and, for instance, in the formulation of the resuspension and deposition processes and the exchanges of solutes and solids. This approach will constitute a significant step in the development of coupled benthic-pelagic biogeochemical models that will allow the modelers to refine the assessment of the role of continental shelves and macrobenthos in the mediation of biogeochemical cycles.

The developed trait distribution models offer the possibility of estimating how the spatial distribution of traits or indicators might change in response to climate change, enabling us to predict potential changes in the ecosystem functions delivered by the macrobenthos under different shared socioeconomic pathways (SSPs). The mapping of traits can support spatial planning and ecosystem-based management by identifying areas where benthic communities deliver key ecological functions or are particularly vulnerable to disturbance^19,58,59^. In the Black Sea, beam trawling has been shown to impact benthic biodiversity negatively, including that of mussel biogenic reefs^60^, highlighting the need to protect those habitats that provide shelter for a large diversity of free-living and sessile epifauna. In addition, immobile suspension-feeder episodic species, such as mussels, may be especially important for maintaining benthic-pelagic fluxes through biodeposition. They should be prioritized for protection because they provide key ecosystem functions and are the most vulnerable to disturbances. Moreover, shallow coastal finer-grained areas with high biomixing and bioirrigation potentials could play a role in enhancing blue carbon sequestration. Given the current gaps in spatial and temporal data for benthic ecosystems, particularly for the Black Sea, there is an urgent need for validated trait-based indicators and high-resolution ecological maps to support targeted conservation strategies and sustainable management of benthic ecosystems.

## Methods

### Study area

The northwestern shelf of the Black Sea is the most productive region of the basin, given the high nutrient input from rivers per unit of the shelf^61^. The pelagic and benthic systems are tightly coupled, with approximately 30% of the shelf-produced primary production degraded in the sediment, accounting for 40% of the shelf oxygen consumption^62^. The shelf acts as a filter for land and river inputs, with about half of the riverine nitrogen input to the shelf lost in the shelf sediment by denitrification and burial^63^.

The northwestern shelf plays a crucial role in ecosystem functioning and biogeochemical budgets at the basin scale; however, the impact of benthic life variability on shelf functions is still poorly understood. The gradients of bathymetry, bottom substratum, and environmental conditions from the coastal area to the shelf break are expected to affect the distribution of benthic functions. For instance, in^41^, we evidenced that the strong gradient of bottom dissolved oxygen from oxic shallow waters to deep euxinic waters (i.e., no oxygen and a high content of hydrogen sulfide), affects the macrozoobenthic biodiversity.

### Data collection

#### Species collection

**Fig. 12:**
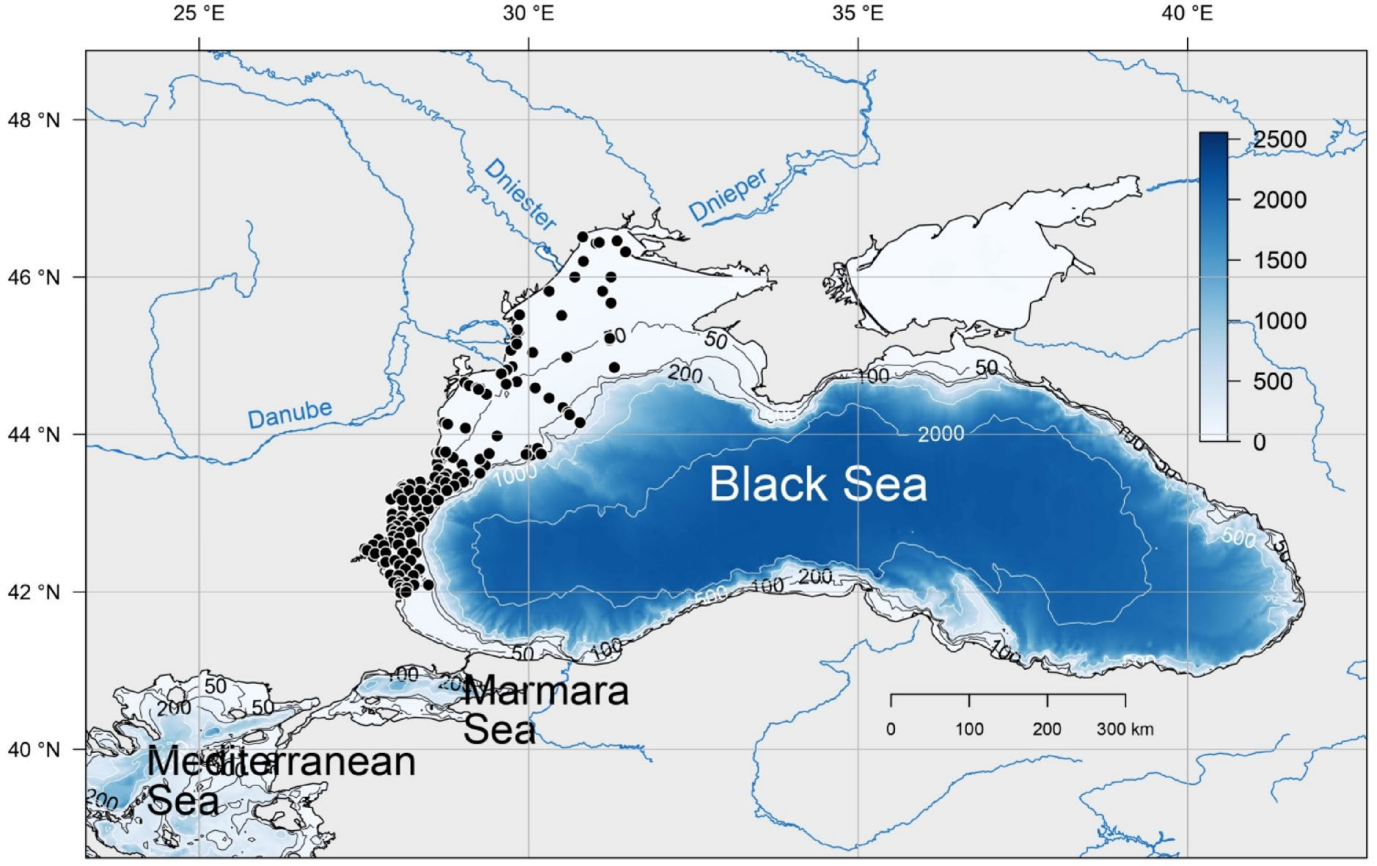
The Black Sea. Main rivers in blue; black dots represent the sampling stations from 2008 to 2017. Contour lines and color bar denote water depth in meters. Map was produced with R.4.3.3^82^. The open-source software is available from https://www.r-project.org/.

We compiled a set of macrobenthos data collected between 2008 and 2017 from 210 sampling stations across the entire northwestern shelf (Fig. 12). Species data have been collected between the start (March) and the end of the stratification period (October). The data were collected according to the standardized macrozoobenthos protocol described in^64^ during all these campaigns. Macrozoobenthic species were sampled with a Van Veen grab with a surface area of 0.135m^2^ and sieved through a 0.5 mm mesh sieve. In the laboratory, the organisms were counted and identified to the lowest possible taxonomic level. Individual organism densities were expressed in number of individuals per m^2^ and were log10 (x + 1) transformed to down-scale large values. More details about the species collection data can be found in^41,65^.

#### Abiotic data

We combined in situ data with data from a model reanalysis delivered by the CMEMS Black Sea Marine Forecasting Center^66^. These data were produced by the coupled hydrodynamical - biogeochemical NEMO4.2-BAMHBI (BiogeochemicAl Model for Hypoxic and Benthic Influenced areas) model run in reanalysis mode at a resolution of 2.5 km with 59 vertical levels. More details on the model can be found in^61,67,68^. We derived the mean, maximum, minimum, and standard deviation for each abiotic variable from a 10-year climatology covering the period of the data collection (i.e., 2008–2018). Coefficients of variation (i.e., the ratio between the standard deviation and the corresponding mean value) were evaluated for temperature and oxygen concentration to assess thermal and oxygen seasonality. Then, we extracted model values at each sampling station (i.e., matrix sampling station × abiotic conditions). Our list of abiotic predictors (Table 1) focuses on a variety of environmental variables expected to influence the distribution of species traits and their derived indicators.

**Table 1:** List of abiotic predictors from the coupled hydrodynamic-biogeochemical NEMO4.2- BAMBHI model or from in situ sampling. The abbreviation and units of each abiotic predictor are shown.

Abiotic descriptors	Abbreviation	Unit
Model variables		
Bottom temperature	TEMP	°C
Coefficient of variation for the temperature	TEMPCV	-
Bottom Salinity	SAL	p.s.u (practical salinity unit)
Bottom shear stress	SHEAR	N m^−2^
Bottom photosynthetically active radiation	PAR	W m^−2^
Bottom Particulate organic carbon content	POC	mmol C m^−3^
Vertically integrated sedimentary organic carbon concentration (fast decay)	fCSED	mmol C m^−2^
Vertically integrated sedimentary organic carbon concentration (slow decay)	sCSED	mmol C m^−2^
Bottom dissolved oxygen concentration	DOX	µmol l^−1^
Coefficient of variation for dissolved oxygen concentration	DOXCV	-
Particulate organic carbon flux to the bottom	botfluxPOC	mmol C m^−2^ day^−1^
In situ **variables**		
In situ depth	Depth	m
Type of substratum	Substratum	Factor with 3 levels: (1) Muddy to sandy mud, (2) Sandy to muddy sand and (3) Coarse and mixed

#### Trait compilation

Among the 206 taxa, we used a subset of 124 taxa for which documentation was available. We selected effect traits with an impact on sediment mixing and nutrients cycling (Table 2) and response traits important for the resilience of benthic communities (Table 4). Each trait was divided into several modalities representing either intervals along a gradient (e.g., lifespan, in years) or qualitative states (e.g., feeding type). When a taxon had clear affinity for one or more modalities of a given trait, it was attributed 1 for the corresponding modalities, 0 everywhere else. When the information was not available at the species level, information at the genus level was considered. More details about trait compilation can be found in^65^.

### Building ecological indicators

#### Indicators based on effect traits

The contribution of each species to an ecological process was estimated via indicators, meaning a trait derived from a formula incorporating several traits^69^. Here, we chose the indicators defined in^54^ for four ecological processes that play a key role in the mediation of benthic-pelagic coupling: biomixing (Eq. 1), bioirrigation (Eq. 2), biodeposition (Eq. 3) and deposit feeding (Eq. 4; Table 3). Each indicator is a combination of traits that have an effect on ecological processes. Within a trait, a score was attributed to each of the modalities, with an increasing score indicating an expected greater effect on ecosystem functioning (Table 2). Then, for each species, indicators were computed as the product of their modality scores following the respective formula given in Table 3. The resulting trait-based indicator was rescaled between 0 and 1 for each species. More details on indicators can be found in^54^.

**Table 2:** The selected effect traits are listed with their units, abbreviations, modalities, descriptions and modality scores that indicate the contribution of the modality to the intensity of the selected ecological processes defined in Table 3.

Trait [Unit] (Abbreviation)	Modality	Description	Modality score
Body Mass [g AFDM] (BM)	< 0.001	Body size, amount of living tissues. Expresses well metabolic demand.	1
	0.001–0.010		2
	0.010–0.100		3
	0.100-1.000		4
	1.000–10.000		5
	> 10.000		6
Feeding type (FT)	Deposit feeding (De)	Informs on food resource origin and how organic matter is processed.	1
	Suspension feeding (Su)		1
	Herbivory/Grazing (HeGr)		1
	Carnivory/Scavenging (CaSc)		1
Substratum depth occupancy [cm] (SD)	0	Occupied layers of the sediment.	1
	0–5		2
	5–15		3
	15–30		4
	> 30		5
Mobility (MB)	Immobile	Dwelling mode, metabolic demand and species interactive potential.	0
	Limited		1
	Slow		2
	Fast		3
	Very fast		4
Sediment mixing type (AF)	None	Type of sediment particle displacement. Diffusion, random, local. Conveying, vertical, non-local. Regeneration, Instantaneous downward transfer (e.g. large burrow collapse).	0
	Diffusion		1
	Upward conveying		1
	Downward conveying		1
	Regeneration		1
Ventilation/Pumping (VP)	Null	Ability to flush water into a burrow.	1
	Low		2
	High		3
Endo-bioconstruction type (BT)	None	Biogenic structure build in the sediment; Closed, only one opening. Opened, U- or Y- shaped burrow, possibly more than two openings.	1
	Blind-ended		2
	Open-ended		3
Endo-bioconstruction depth [cm] (maxBD)	None/Surficial	Size of endo-bioconstruction (e.g., depth of a burrow).	0
	0–5		1
	5–15		2
	15–30		3
	> 30		4

**Table 3:** Indicator of ecological process at the species level with their definition and formula. Details on each trait used the in formula can be found in Table 2.

Indicator	Definition	Formula
Biomixing (mi)	One of the two processes of bioturbation through particle reworking^4^.	mi = AF × BM × MB × SD (Eq. 1)
Bioirrigation (ir)	One of the two processes of bioturbation through ventilation^4^.	ir = VP × BM × BT × maxBD (Eq. 2)
Biodeposition (de)	Benthic-pelagic transfer through suspension feeding^8^.	de = BM × Su (Eq. 3)
Deposit feeding (Dep)	Ingestion of large volumes of sediment^10^	Dep = BM × De (Eq. 4)

#### Indicators based on response traits

Life history strategies involve species that have similar abilities to withstand mortality at juvenile and adult stages based on the POSE concept, which differentiates four main groups: **P**recocial, **O**pportunist, **S**urvivor and **E**pisodic^36^. Briefly, a first axis separates fast (precocial and opportunist) and slow (survivor and episodic) life history strategies, and the second axis separates high (opportunist and episodic) and low (precocial and survivor) juvenile mortality rates.

In our study, each species was assigned to one of the four life history strategies, based on their trait modalities (Table 4). Firstly, a fuzzy correspondence analysis (FCA) was applied to the trait matrix. Then, life history groups were derived using hierarchical clustering. Ward’s aggregation criterion^70^ was applied to species scores from the first two axes of the FCA. More details on the methodology can be found in^39^. In the Supplementary Information, more detailed results about the FCA and clustering can be found.

**Table 4:** The selected response traits are listed with their units, modalities and descriptions.

Trait [Unit]	Modality	Description
Life span [years]	< 1	Time necessary to achieve a life cycle during which at least one reproductive success is ensured; informs also on growth rate.
	1–3	
	3–10	
	10–20	
	> 20	
Age at maturity [years]	< 1	Time after which reproductive success can be expected; informs also on growth rate.
	1–3	
	> 3	
Sexuality	Gonochorism	Gonochorism, constraint of reproductive completion, unlike homogamy. Protandry, growth constraint.
	Homogamy	
	Protandry	
Reproductive frequency	Sexual seasonal	Seasonal reproduction only during specific times of the year, while continuous reproduction is throughout the year.
	Sexual continuous	
	Asexual	
Fertilization	Broadcasting	Informs on proximity between genitors.
	Spermcasting	
	Pairing	
Annual fecundity [number of offsprings]	< 10e2	Potential of annual demographic recruitment. Generally, correlated to offspring mortality.
	10e2-10e3	
	10e3-10e4	
	10e4-10e5	
	10e5-10e6	
	> 10e6	
Offspring type	Egg	Offspring once released and independent from the parents. Expresses offspring survival.
	Larva	
	Juvenile	
Offspring size [mm]	< 0.1	Reproductive allocation per capita.
	0.1–0.5	
	0.5–1.5	
	> 1.5	
Offspring protection	None	Expresses parental cares and offspring survival.
	Gel	
	Capsule	
	Bearing/Brooding	
Offspring development	Planktotrophic	Informs on developmental complexity, embryonic vulnerability and adult reproductive effort.
	Lecithotrophic	
	Mixed planktotrophic	
	Mixed lecithotrophic	
	Internal	
Offspring benthic stage duration [days]	Null	Critical time on the sea floor necessary to achieve offspring development.
	< 15	
	15–30	
	30–60	
	> 60	
Offspring pelagic stage duration [days]	Null	Critical time in the water column necessary to achieve offspring development.
	< 15	
	15–30	
	30–60	
	> 60	
Offspring settlement size	< 0.5	Early body size. See below.
	0.5–1.5	
	> 1.5	
Body mass [g AFDM]	< 0.001	Body size, amount of living tissues. Expresses well metabolic demand.
	0.001–0.010	
	0.010–0.100	
	0.100-1.000	
	> 1.000	
Body length [cm]	< 1	Length of the main body part, not necessarily correlated to body mass. Strongly involved in space occupation, also in vulnerability to predation.
	1–3	
	3–10	
	10–20	
	> 20	
Mobility	Immobile	Dwelling mode, metabolic demand and species interactive potential.
	Limited	
	Slow	
	Fast	
	Very fast	
Substratum depth occupancy [cm]	0	Occupied layers of the sediment.
	0–5	
	5–15	
	15–30	
	> 30	
Feeding type	Deposit feeding	Informs on food resource origine and how organic matter is processed.
	Suspension feeding	
	Herbivory/Grazing	
	Carnivory/Scavenging	

### From species to community scale

#### Community weighted-mean traits

The community weighted mean of a trait (CWM) is a weighted average of the trait modalities of all the species at the scale of a community^71^. For response traits, we used relative mean values (i.e., species densities relative to the total density of the community). For effect traits, we used the absolute means of a trait, as an expected greater effect on ecosystem functions is expected with higher individual densities.

#### Community-level indicators

The community-level potentials for biomixing, bioirrigation, biodeposition, and deposit feeding were calculated by summing, across all species, the product of each species density by the species score. The community level potential is then scaled to be between 0 and 1. For life history groups, species densities were expressed as the ratio of density to the total density of a site. Then, relative species densities are summed up by group within each community.

### ﻿﻿﻿﻿From community to ecosystem scale﻿﻿﻿

**Fig. 13:**
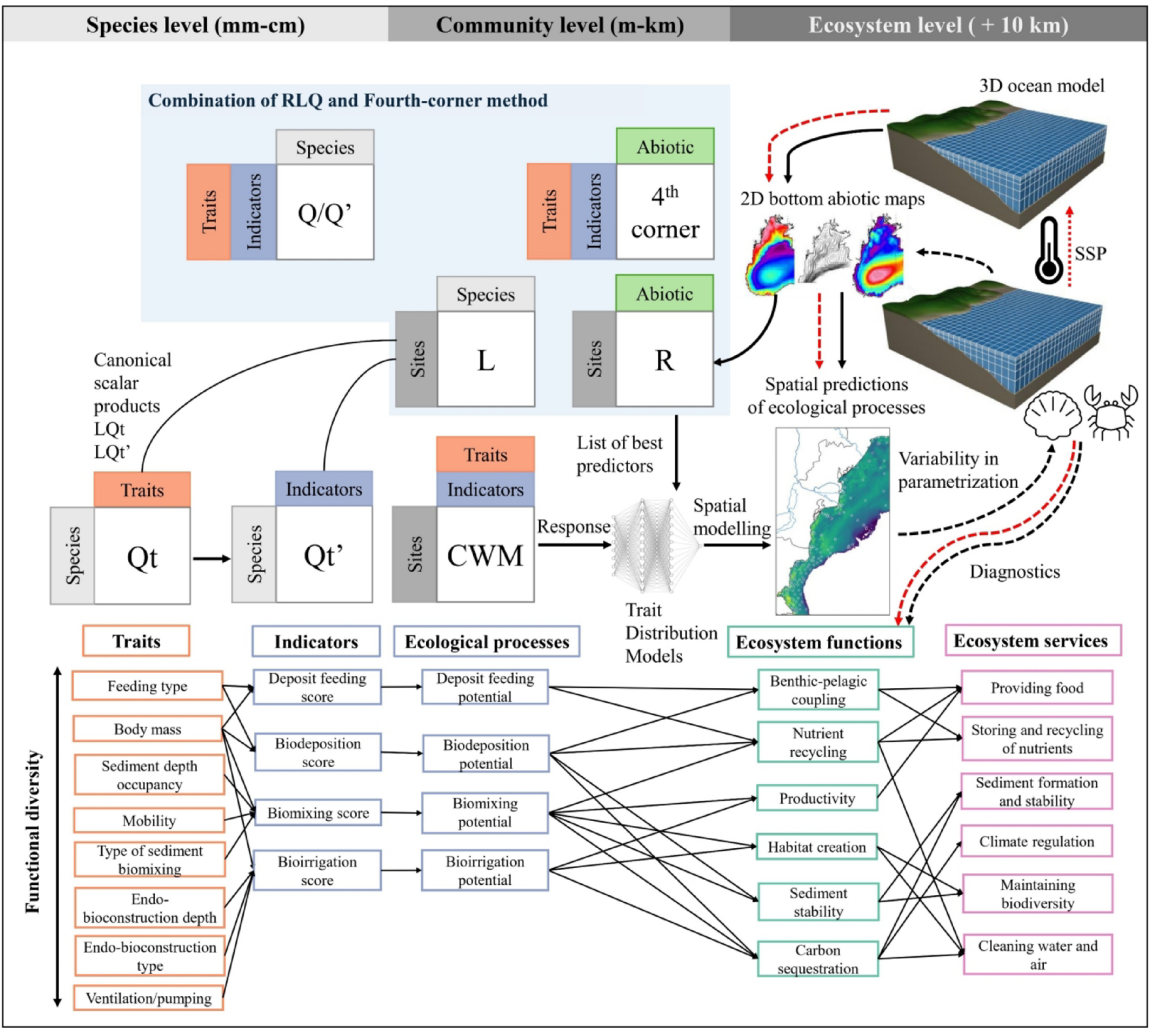
Upscaling from the species level to ecosystem services. The list of effect traits considered in this study related to ecological processes at the community scale and to ecosystem functions and ecosystem services at a larger scale. This paper mainly focuses on the link from species traits to the spatial distribution of ecological processes at the shelf scale (i.e., maps). Here, black dashed arrows indicate the potential question of research, and red dashed arrows indicate projections in the context of global change. Figure adapted from^20^. Model grid from https://www.comet.ucar.edu/.

**Fig. 14:**
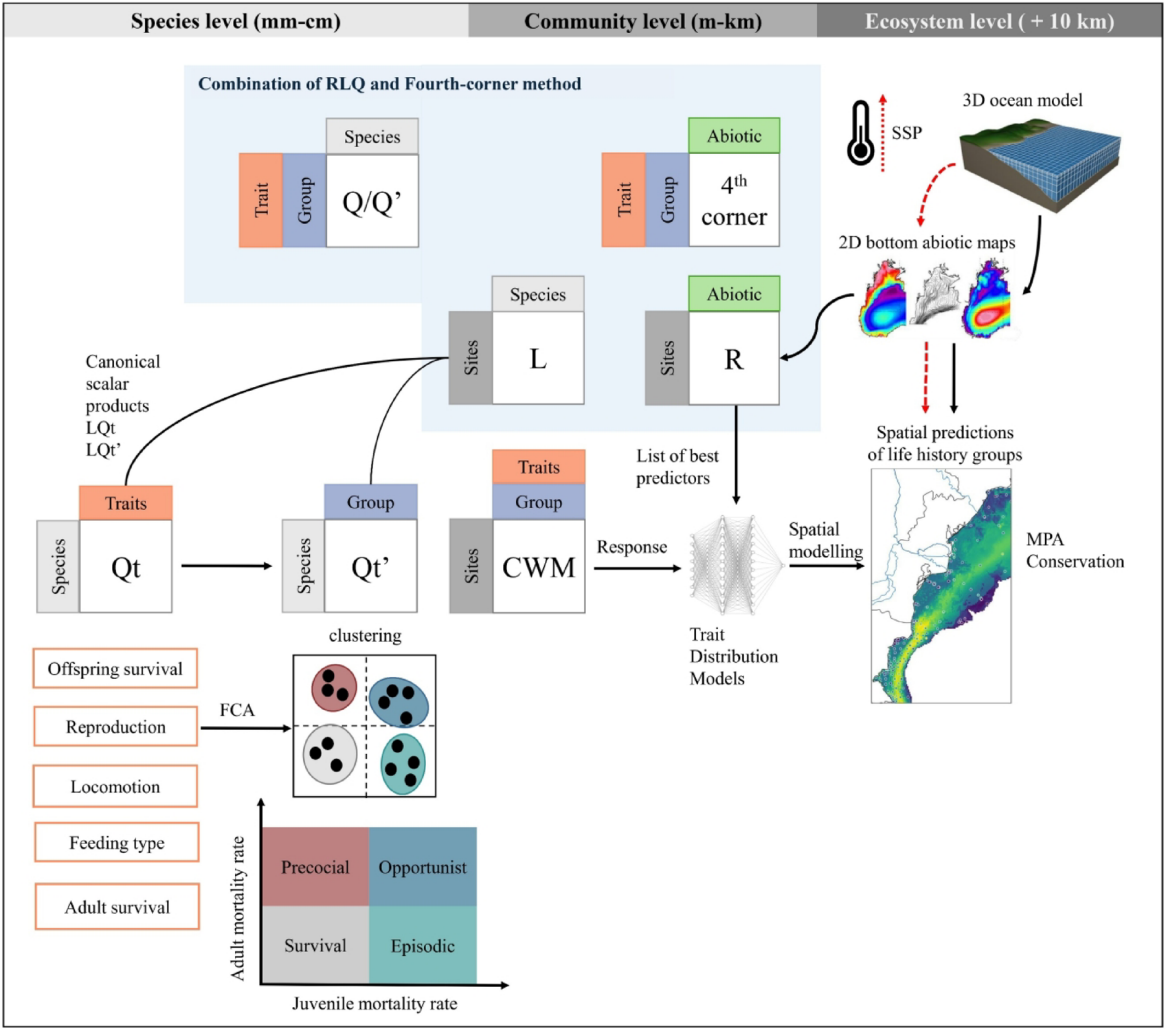
Upscaling from the species level to the maps of life history strategies for conservation and marine protection area (MPA) management. The definition of life history groups is based on the concept “POSE”^36^. Here, the red dashed arrows represent projections in the context of global change. Figure adapted from^20^. Model grid from https://www.comet.ucar.edu/.

The upscaling from the community level up to the ecosystem was done using trait distribution models (TDMs). The trait-environment relationships were identified at the local scale, and then, using environmental conditions delivered by a reanalysis (CMEMS here), the traits were mapped at shelf scales using a neural network. The methodology used to investigate local trait-environment relationships is first described below, along with the neural network and spatial prediction. We provided a summary of our methodology for both ecological processes and life history strategies in Figs. 13 and 14, respectively.

#### Local trait-environment relationships

Our data set is composed of three matrixes: a matrix (R) that contains the sampling stations (rows) and the abiotic descriptors (columns); a matrix (L) that contains the sampling stations (rows) and the taxa matrix (columns); and a matrix (Q) that contains the taxa (rows) and the traits or indicators (columns). We used an RLQ analysis in combination with the fourth-corner approach to identify the environmental variables that are significantly correlated with the traits or indicators^72,73^.

Before the RLQ analysis, an exploratory analysis was performed to select the most relevant environmental descriptors and traits. Traits and abiotic variables were tested according to the fourth-corner method combined with RLQ^72^. This preliminary procedure is used to increase the global significance of trait-environment relationships. We kept the abiotic factors likely correlated with the trait matrix and the traits likely correlated with the abiotic data. We provided the results of preliminary tests in the Supplementary Information. After an appropriate selection of traits and environmental descriptors, we applied an RLQ analysis with the new tables (reduced) R and Q. As a first step, we applied separate univariate analyses on the three tables: a multiple correspondence analysis (MCA) on environmental table R (using qualitative abiotic data as in^39,41^); a correspondence analysis (CoA) on the species table L; and a fuzzy correspondence analysis (FCA) on the fuzzy-coded trait data table (Q) or principal component analysis (PCA) if traits are quantitative. Separate analyses of traits and environmental variables were weighted respectively by the species and site weights derived from the CoA^74^. The three separate analyses were subsequently combined in the RLQ analysis framework. The degree of associations between trait and environmental tables was quantified by RLQ inertia^75^. The significance of the total inertia of the RLQ analysis was tested by 49 999 row permutations of table L to break with the link with Table R (i.e., Model 2) and column permutations to break the link with table Q (i.e., Model 4). Relationships were significant when the p values of both model tests were lower than the fixed threshold of 0.05^76^. A p value adjustment was performed following the false discovery rate method^77^. Then, we tested the correlation between the RLQ axes and individual traits or abiotic conditions, as proposed in^72^. Directly testing the associations between RLQ axes and traits/environmental variables improved the interpretability of the RLQ results. The RLQ and fourth-corner analyses were performed using the ade4 package for R software^74^.

#### Trait distribution modeling

Based on the general trend derived from multivariate analysis, we adapt trait distribution models (hereafter TDMs) to detect local relationships between community weighted-mean indicator values and a suite of abiotic descriptors measured at the same sampling station. In our study, we chose to use a neural network for our TDMs, in response to a growing interest in machine learning tools in ecology and their ability to resolve complex spatial and temporal nonlinear patterns in oceanography^78,79^. We used the scikit-learn library for the Python programming language^80^.

To generate spatial maps of traits, based on discrete data at a limited number of stations, we used a feedforward neural network comprising an input layer, 2 hidden layers with 12 neurons each, using a leaky ReLU activation function, and an output layer, leading to 601 trainable parameters. The method is implemented using Keras software with the Torch backend. The hyperparameters are tuned by splitting the station data into a training set (50% of the data), a validation set (30%) and a test set (20%), and repeating the training 50 times. The score is the mean squared error between the real output and the one predicted by the neural network. Once trained, the network is then applied to each pixel of the domain to infer the traits. The input data consists of the bottom type (mud, sand, or gravel), the depth, climatological, standard-deviation, minimum and maximum for the variables listed in Table 1, obtained from the Copernicus Marine Black Sea Reanalysis (10.25423/CMCC/BLKSEA_MULTIYEAR_BGC_007_005_BAMHBI) and computed using the coupled NEMO-BAMHBI physical-biogeochemical model. All the inputs are normalized.

## Supplementary Information

Below is the link to the electronic supplementary material.

Supplementary Material 1

## Data Availability

Traits and species data are accessible from the repository *Figshare* (accessible online here: [https://figshare.com/s/328e4e6feae73718b5a5] (https:/figshare.com/s/328e4e6feae73718b5a5) ), and the data descriptor paper is freely accessible in *Scientific Data* ( [https://doi.org/10.1038/s41597-025-05311-2] (https:/doi.org/10.1038/s41597-025-05311-2) ). Data sets can be freely viewed and downloaded from *Figshare* and users are required to cite this data paper in any resulting work (license CC-BY).
