# Supplementary material for: Trait modeling to predict benthic functions and vulnerabilities across black sea seascapes
Source: Sci Rep. 2025 Nov 7;15:39076. doi: 10.1038/s41598-025-24508-4 (PMC12594983; doi:10.1038/s41598-025-24508-4)
Supplement: Supplementary file 1 — Supplementary Material 1 [file 41598_2025_24508_MOESM1_ESM.pdf]

Supplementary Information

Supplementary Information 1
1. Definition of life history groups 1
2. RLQ analysis details 5
2.1 Life history groups 5
2.2 Response traits 7
2.3 Effect traits 10
2.4 Ecological processes 12

1. Definition of life history groups

Fig. S1 presents the ordination of species based on their response traits. Three main groups of life history are derived: “O”- opportunistic (dark blue), “E”-episodic (dark cyan) and “P”-precocial (dark red). The first axis separates the “P” and “E” strategies, and the second axis separates the “O”-strategists from the “P” and “E”-strategists (Fig. S1).

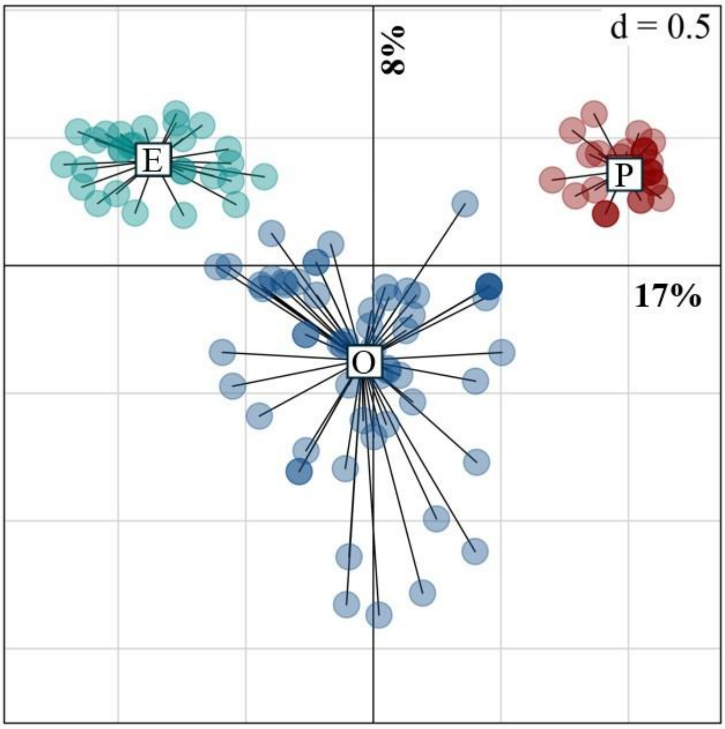

Fig. S1 Ordination of species based on their response traits using a fuzzy correspondence analysis (FCA). Species scores on the first axis (x) and second axis (y) of the FCA. Species are represented by dots; colored dots correspond to a life history strategy (dark red: “Precocial”, dark blue: “Opportunist” and dark cyan = “Episodic”). Size of the grid in the top-right corner. The first two axes of the fuzzy correspondence analysis (FCA) explained 25% of the total inertia, with 17% for the first and 8% for the second axes.

The traits contributing most to the axes were identified by their correlation ratios (CR, Table S1). Traits linked to reproductive (e.g., annual fecundity) and offspring characteristics (e.g., offspring size, protection, development) discriminated the most species according to their differences in traits.

Table S1. Correlation ratios (CR) between the first and second axes of the FCA and functional traits. CR returns the proportion of explained variance of the axis species scores by the discrimination of the trait modalities.

	CR - axis 1	CR - axis 2
Life span	0.44	0.07
Age at maturity	0.34	0.04
Sexuality	0.00	0.34
Reproductive frequency	0.33	0.03
Fertilization	0.62	0.50
Annual fecundity	0.72	0.25
Offspring type	0.72	0.52
Offspring size	0.75	0.41
Offspring protection	0.66	0.12
Offspring development	0.87	0.74
Offspring benthic stage duration	0.63	0.21
Offspring pelagic stage duration	0.67	0.22
Offspring settlement size	0.44	0.02
Body mass	0.30	0.08
Body length	0.44	0.08
Mobility	0.27	0.29
Substratum depth occupancy	0.11	0.06
Feeding type	0.24	0.04

Episodic species are generally large, long-lived, and have late sexual maturity. These taxa lack parental care and often reproduce through seasonal broadcasting of numerous small, planktotrophic pelagic eggs, which are unprotected and have high mortality rates (Fig. S2). Bivalves are representative of this group with other phylogenetically distant taxa, such as echinoderms and polychaetes (Fig. S3). The opportunist group is in a central position with a lower adult survival rate than the episodic group but with a higher juvenile survival rate (closer to the precocial group). This group is both phylogenetically and functionally diverse (Figs. S2 and S3). The precocial group primarily includes small species with short life spans and early sexual maturity (Fig. S2). These species produce a small number of large benthic juvenile offspring after internal incubation, often in a continuous reproductive mode (Fig. S2). These biological traits are strongly specific to amphipods and cumaceans (Fig. S2).

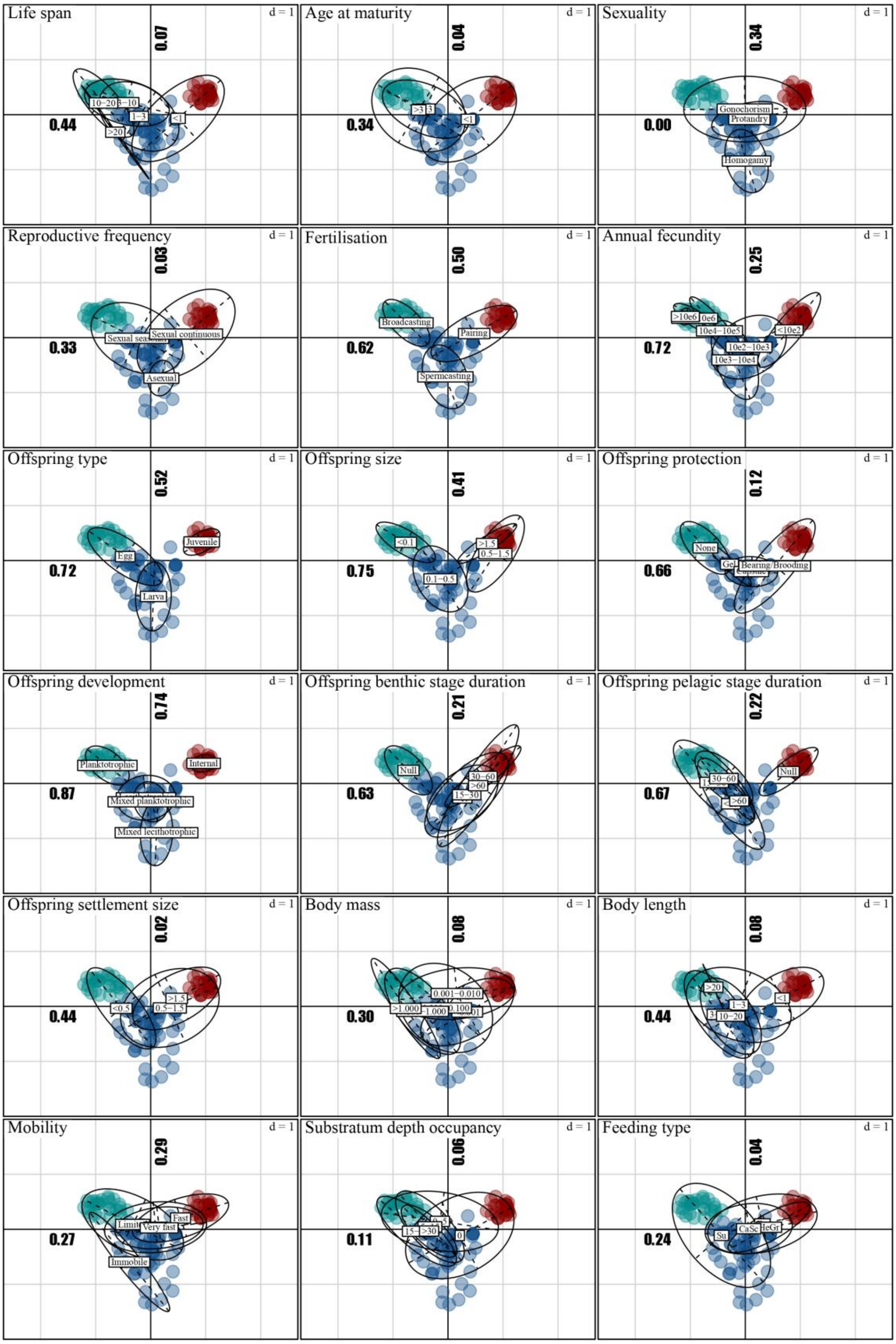

Fig. S2 Distribution of trait modalities (ellipse) for each trait (windows). Taxa are represented by colored dots; each color corresponds to a life history group with dark red for “Precocial”, dark blue for “Opportunist” and dark cyan for “Episodic”. The respective correlation ratios (CR) for each trait for the first and second axes are in bold (Table S1). The trait modalities are positioned at the center of gravity of their respective taxa. Size of the grid in the top-right corner.

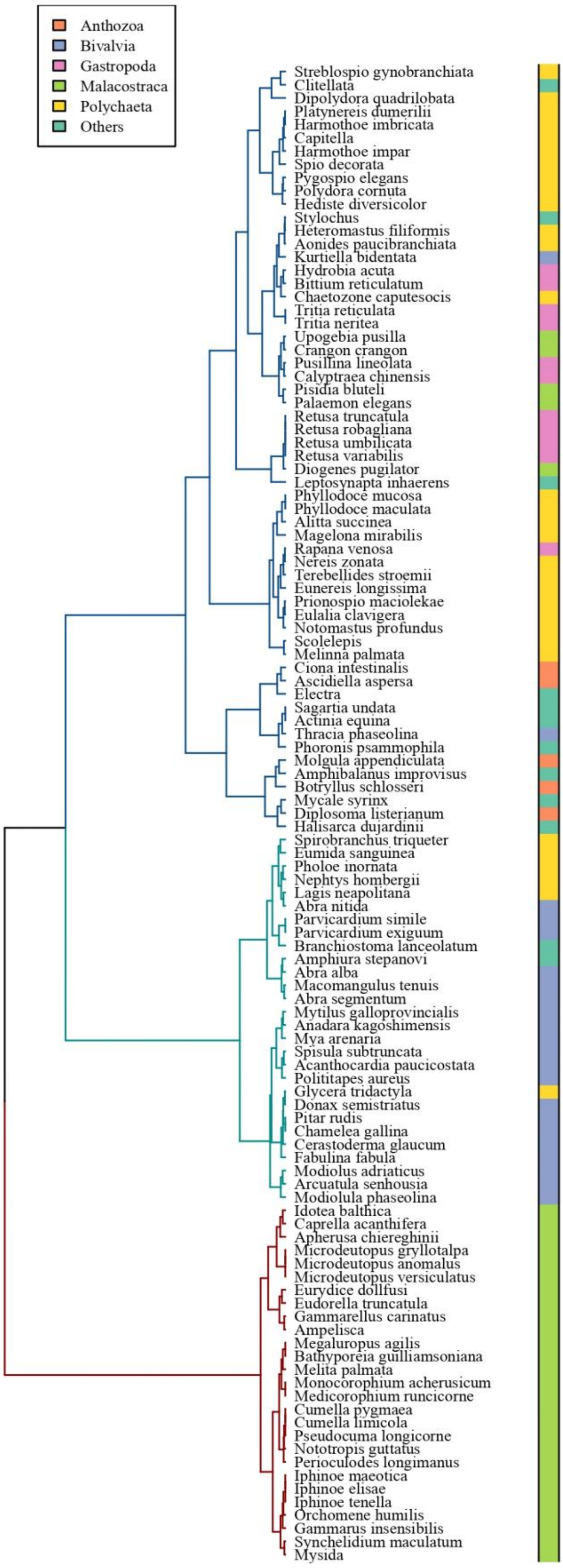

Fig. S3 Main groups of life history : “Precocial” (dark red), “Opportunist” (dark blue) and “Episodic” (dark cyan) species. On the right, the color bar corresponds to the taxon class, with the legend in the top right corner (class level, others merge classes for which the number of species was less than five).

2. RLQ analysis details

2.1 Life history groups

2.1.1 Preliminary test

Table S2. Results of tests for multivariate statistics. Tests between individual environmental conditions and life history groups. The selected variables are in bold and marked by an asterisk. The simulated statistics were generated by 999 random permutations.

Abiotic	Coinertia	p value
DOX	0.03	0.57
SHEAR	0.02	0.46
botfluxPOC	0.02	0.39
SAL	0.03	0.43
TEMP	0.01	0.85
**POC**	**0.04**	**0.29***
PAR	0.01	0.76
FSED	0.01	0.78
SSED	0.02	0.47
Depth	0.03	0.48
Substratum	0.01	0.51
TEMPCV	0.02	0.60
DOXCV	0.01	0.57

2.1.2 RLQ analysis

Table S3. Models 2 and 4 observations and their associated p values.

Test	Obs	Std. Obs	p value
Model 2	0.023	15.859	0.001***
Model 4	0.023	0.313	0.310

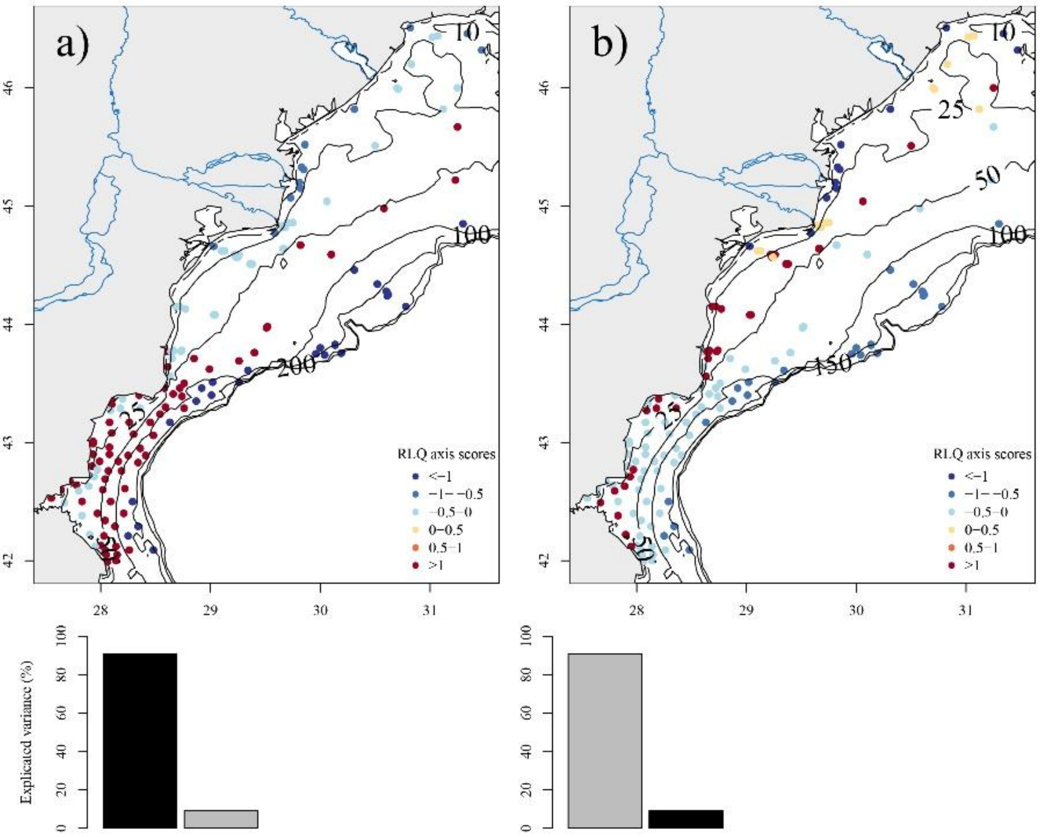

Fig. S4 a) Sampling station scores on the first axis of the RLQ analysis (from dark blue negative scores to red positive scores on the first axis), explained variance (%) by the first axis (91%) and b) second axis (9%) of the RLQ analysis. Map was produced with R.4.3.3. The open-source software is available from https://www.r-project.org/.

2.1.3 Fourth-corner in combination with RLQ

Table S4. Combination of fourth-corner and RLQ results. A) Fourth-corner tests between the overall RLQ and the first two RLQ axes for the environmental gradient and traits. B) Fourth-corner tests between the overall RLQ and the first two RLQ axes for trait syndromes and environmental variables. Significant associations are in green (p value < 0.05).

A) Abiotic	coinertia	Axis 1	coinertia	Axis 2	coinertia	Axis 1:2
POC	0.029	0.071	0.003	0.83	0.032	0.231
B) Group	coinertia	Axis 1	coinertia	Axis 2	coinertia	Axis 1:2
Episodic	0.001	0.383	0.0004	0.791	0.001	0.638
Opportunist	0.009	0.006**	0.0002	0.791	0.009	0.075
Precocial	0.009	0.006**	0.0020	0.791	0.011	0.060

2.2 Response traits

2.2.1 Preliminary test

Table S5. Results of tests for multivariate statistics. Tests between individual environmental conditions and response traits. The selected variables for the RLQ analysis are in bold and marked by an asterisk (p value < 0.10). The simulated statistics were generated by 999 random permutations.

	Coinertia	p value
**DOX**	**0.93**	**0.08***
SHEAR	0.45	0.41
botfluxPOC	0.50	0.37
**SAL**	**0.77**	**0.05***
TEMP	0.56	0.26
POC	0.82	0.20
PAR	0.45	0.11
FSED	0.54	0.18
SSED	0.72	0.13
Depth	0.93	0.15
Substratum	0.39	0.42
TEMPCV	0.69	0.33
DOXCV	0.38	0.48

Table S6. Results of tests for multivariate statistics. Tests between response traits and previously selected abiotic descriptors. The selected variables for the RLQ analysis are marked by an asterisk (p value < 0.05). The simulated statistics were generated by 999 random permutations.

	Coinertia	p value
Life span	0.096	0.263
Age at maturity	0.023	0.856
**Sexuality**	**0.258**	**0.002***
Reproductive frequency	0.104	0.091
Fertilization	0.027	0.859
Annual fecundity	0.048	0.963
Offspring type	0.037	0.679
Offspring size	0.055	0.642
Offspring protection	0.129	0.087
**Offspring development**	**0.155**	**0.041***
Offspring benthic stage duration	0.109	0.236
Offspring pelagic stage duration	0.105	0.272
Offspring settlement size	0.096	0.105
Body mass	0.065	0.706
Body length	0.118	0.143
**Mobility**	**0.177**	**0.024***
Substratum depth occupancy	0.062	0.582
Feeding type	0.036	0.839

2.2.2 RLQ analysis

Table S7. Models 2 and 4 observations and their associated p values.

Test	Obs	Std. Obs	p value
Model 2	0.057	40.715	0.00002***
Model 4	0.057	4.797	0.00052***

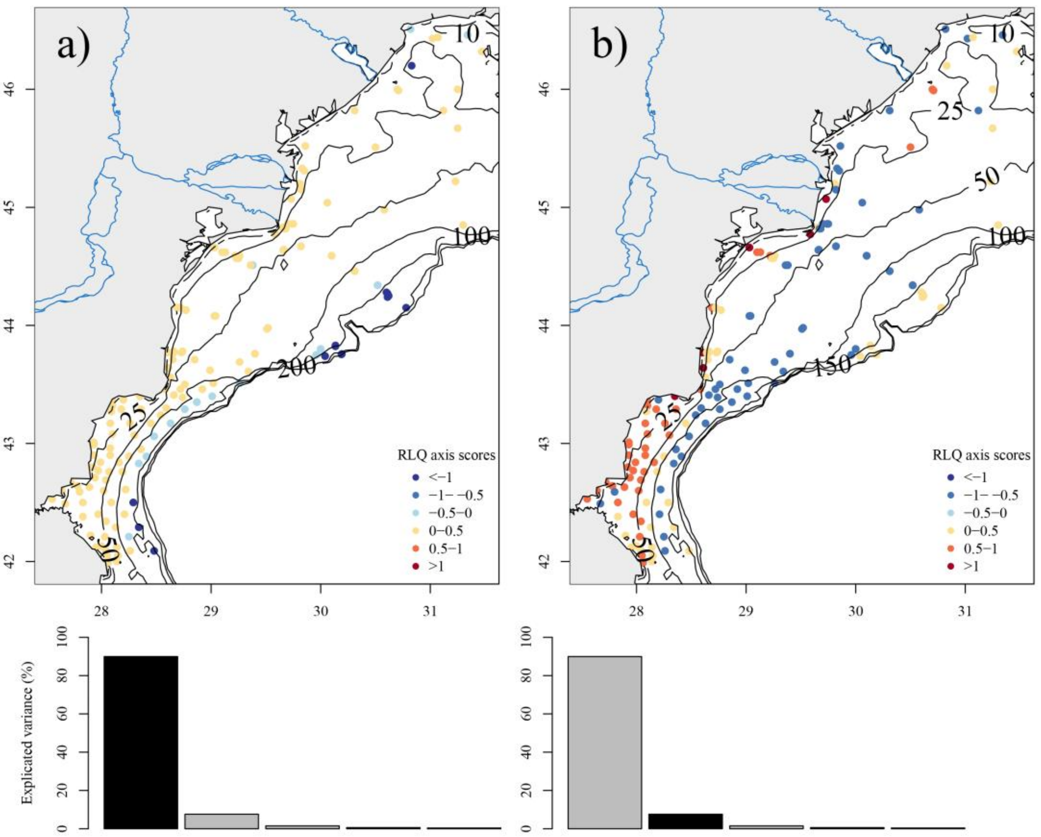

Fig. S5 a) Mapping of the scores of the sampling sites on the first axis of the RLQ analysis and b) second axis. Bottom line. The eigenvalues of the first and second axes are expressed as percentages, with 90% for the first axis and 8% for the second axis. Map was produced with R.4.3.3. The open-source software is available from https://www.r-project.org/.

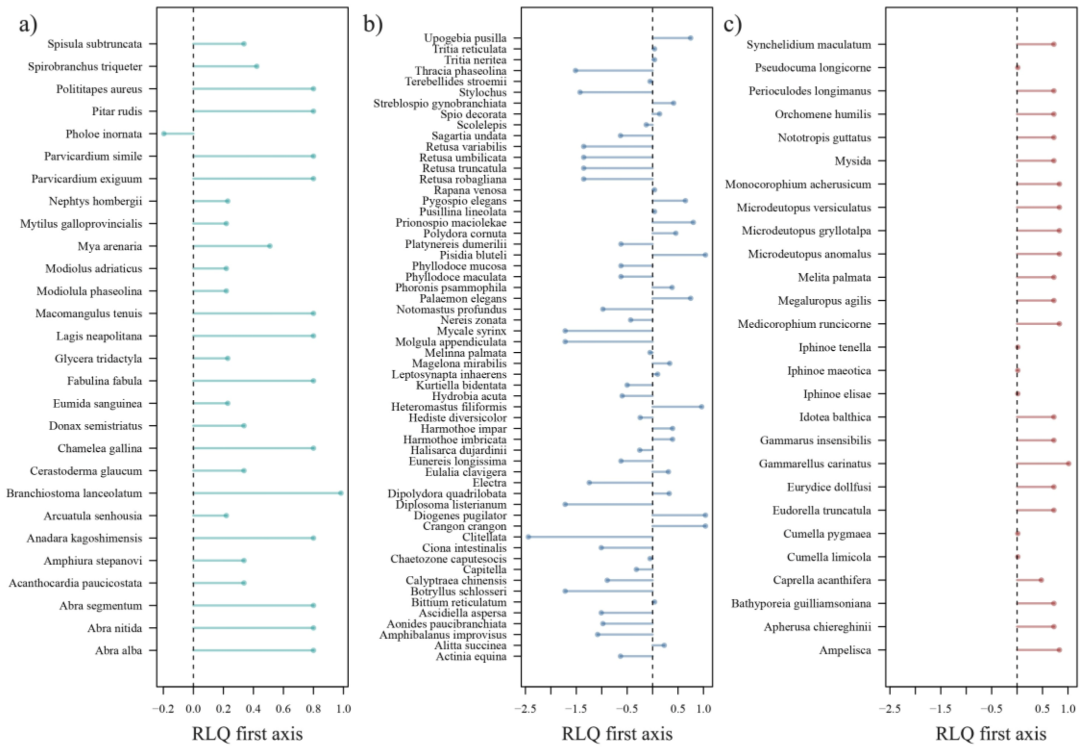

Fig. S6 Species scores per life history strategy on the first axis of the RLQ: a) "Episodic", b)"Opportunist" and c) "Precocial" species.

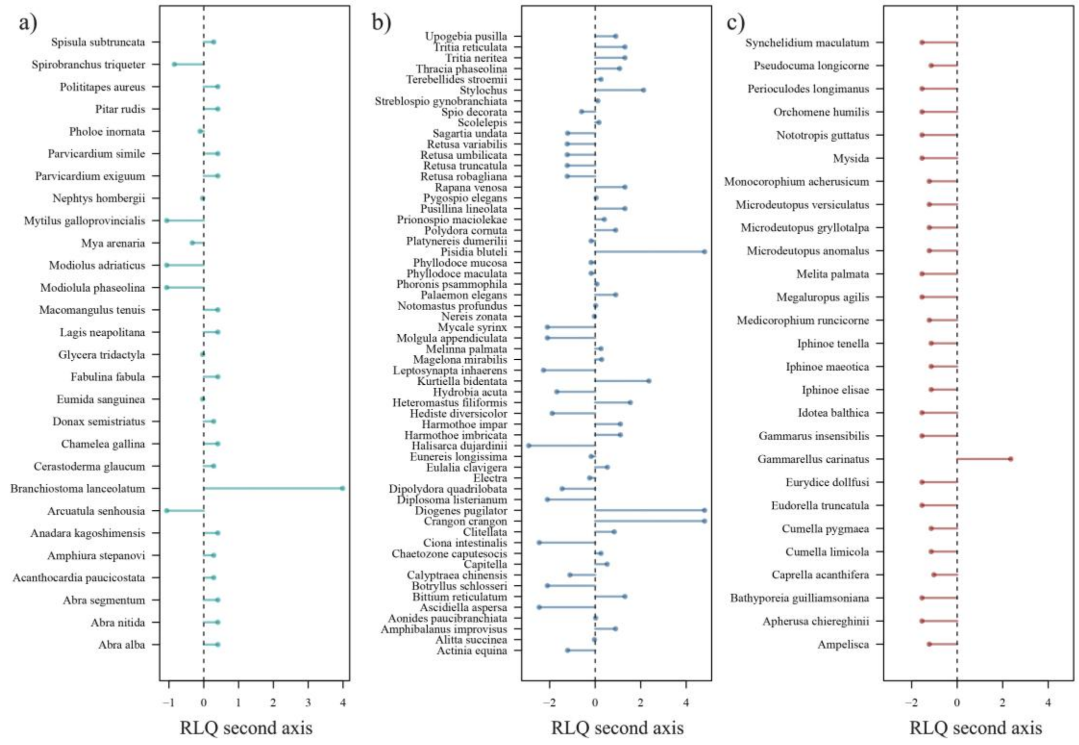

Fig. S7. Species scores per life history strategy on the second axis of the RLQ: a) "Episodic", b)"Opportunist" and c) "Precocial" species.

2.2.3 Fourth-corner in combination with RLQ

Table S8: Combinations of fourth-corner and RLQ results. A) Fourth-corner tests between the overall RLQ and the first two RLQ axes for the environmental gradient and traits. B) Fourth-corner tests between the overall RLQ and the first two RLQ axes for trait syndromes and environmental variables. Significant associations are in green (p value < 0.05).

A) Abiotic	coinertia	Axis 1	coinertia	Axis 2	coinertia	Axis 1:2
DOX	0.102	0.00004***	0.031	0.133	0.133	0.00012***
SAL	0.145	0.00004***	0.010	0.396	0.155	0.00020***
B) Indicator	coinertia	Axis 1	coinertia	Axis 2	coinertia	Axis 1:2
Sexuality	0.130	0.007**	0.003	0.613	0.134	0.009**
Offspring development	0.068	0.048*	0.015	0.613	0.083	0.038*
Mobility	0.083	0.032*	0.011	0.613	0.093	0.035*

2.3 Effect traits

2.3.1 Preliminary test

Table S9. Results of tests for multivariate statistics. Tests between individual environmental conditions and traits. The selected variables for the RLQ analysis are in bold and marked by an asterisk (**i.e., p value < 0.10**). The simulated statistics were generated by 999 random permutations.

	Coinertia	p value
DOX	0.128	0.458
SHEAR	0.175	0.213
botfluxPOC	0.209	0.09
**SAL**	**0.082**	**0.017***
TEMP	0.231	0.220
POC	0.230	0.290
PAR	0.106	0.441
FSED	0.125	0.416
SSED	0.142	0.578
Depth	0.321	0.151
**Substratum**	**0.235**	**0.067***
TEMPCV	0.235	0.382
DOXCV	0.087	0.184

Table S10. Results of tests for the multivariate statistic. Tests between individual traits and previously selected abiotic descriptors. The selected variables for the RLQ analysis are marked by an asterisk (**i.e., p value < 0.05**). The simulated statistics were generated by 999 random permutations.

	Coinertia	p value
BM	0.031	0.277
MB	0.018	0.582
**SD**	**0.046**	**0.057***
BT	0.018	0.266
maxBD	0.030	0.262
**VP**	**0.045**	**0.021***
AF	0.038	0.107
FT	0.009	0.818

2.3.2 RLQ analysis

Table S11. Models 2 and 4 observations and their associated p values.

Test	Obs	Std. Obs	p value
Model 2	0.014	29.405	0.00002***
Model 4	0.014	0.903	0.14

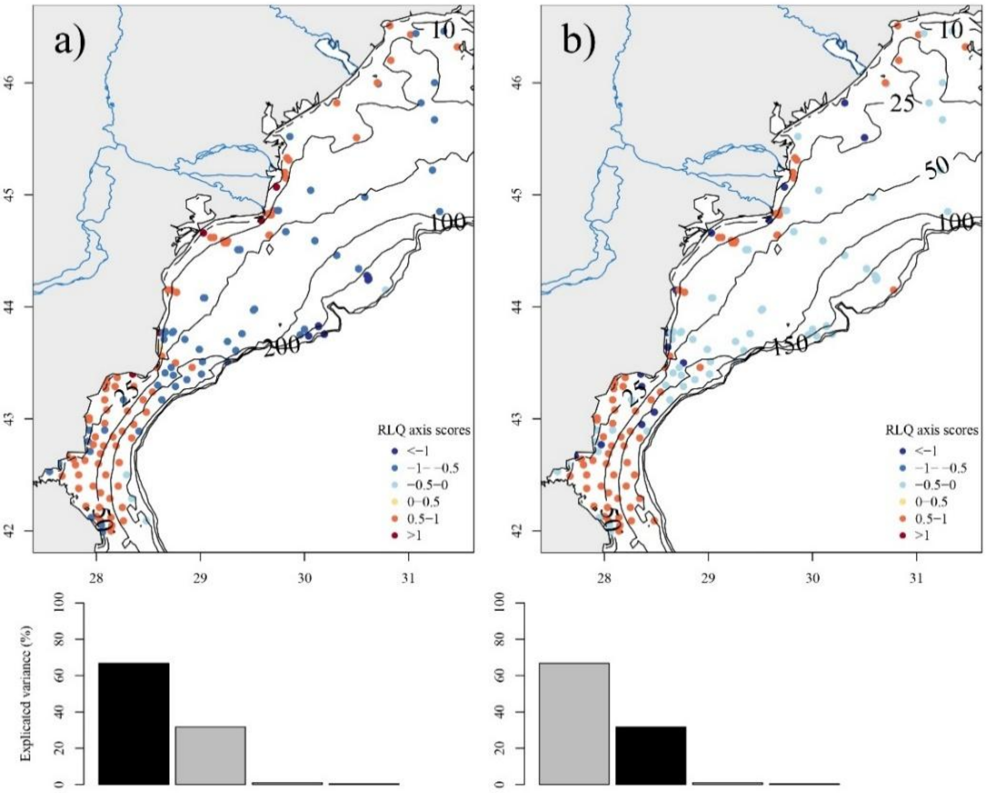

Fig. S8 a) Mapping of the scores of the sampling sites on the first axis of the RLQ analysis and b) second axis. Bottom line. The eigenvalues of the first and second axes are expressed as percentages, with 67% for the first axis and 32% for the second axis. Map was produced with R.4.3.3. The open-source software is available from https://www.r-project.org/.

2.3.3 Fourth-corner in combination with RLQ

Table S12. Combination of fourth-corner and RLQ results. A) Fourth-corner tests between the overall RLQ and the first two RLQ axes for the environmental gradient and traits. B) Fourth-corner tests between the overall RLQ and the first two RLQ axes for trait syndromes and environmental variables. Significant associations are in green (p value < 0.05).

A) Abiotic	coinertia	Axis 1	coinertia	Axis 2	coinertia	Axis 1:2
SAL	0.004	0.144	0.009	0.018*	0.013	0.016*
Substratum	0.048	0.012**	0.019	0.077	0.068	0.016*
B) Indicator	coinertia	Axis 1	coinertia	Axis 2	coinertia	Axis 1:2
Substratum depth occupancy	0.043	0.030*	0.006	0.484	0.049	0.037*
Ventilation/pumping	0.028	0.030*	0.021	0.016*	0.048	0.024*

2.4 Ecological processes

2.4.1 Preliminary test

Table S13. Results of tests for multivariate statistics. Tests between individual environmental conditions and ecological process indicators. The selected variables are in bold and marked by an asterisk (**i.e., p value < 0.1**). The simulated statistics were generated by 999 random permutations.

	Coinertia	p value
DOX	0.01	0.49
SHEAR	0.01	0.51
botfluxPOC	0.08	0.37
**SAL**	**0.01**	**0.10***
TEMP	0.03	0.24
POC	0.01	0.76
PAR	0.01	0.40
FSED	0.01	0.69
SSED	0.00	0.99
Depth	0.04	0.24
Substratum	0.02	0.44
TEMPCV	0.02	0.51
DOXCV	0.01	0.59

Table S14. Results of tests for multivariate statistics. Tests between ecological process indicators and previously selected abiotic descriptors. statistics were generated by 999 random permutations.

	Coinertia	p value
de	0.0005	0.69
mi	0.002	0.17
ir	0.003	0.07
De	0.003	0.13

2.4.2 RLQ analysis

Table S15. Models 2 and 4 observations and their associated p values.

Test	Obs	Std. Obs	p value
Model 2	0.006	6.245	0.002***
Model 4	0.006	1.717	0.064

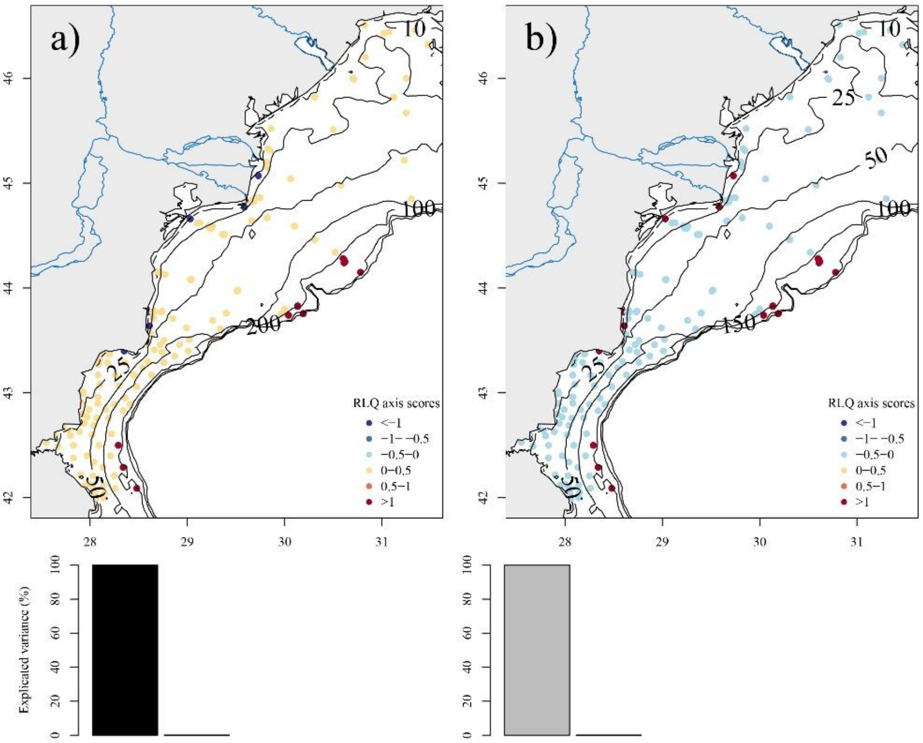

Fig. S9 a) Sampling station scores on the first axis of the RLQ analysis (from dark blue negative scores to red positive scores on the first axis). explained variance (%) by the first axis (99.9%) and b) second axis (9%) of the RLQ analysis. Map was produced with R.4.3.3[82]. The open-source software is available from https://www.r-project.org/.

2.4.3 Fourth-corner in combination with RLQ

Table S16. Combination of fourth-corner and RLQ results. A) Fourth-corner tests between the overall RLQ and the first two RLQ axes for the environmental gradient and traits. B) Fourth-corner tests between the overall RLQ and the first two RLQ axes for trait syndromes and environmental variables. Significant associations are in green (p value < 0.05).

A) Abiotic	coinertia	Axis 1
Salinity	0.006	0.026*
B) Indicator	coinertia	Axis 1
Deposit feeding	0.003	0.047*
Bioirrigation	0.003	0.047*
